# Experimental characterization of de novo proteins and their unevolved random-sequence counterparts

**DOI:** 10.1038/s41559-023-02010-2

**Published:** 2023-04-06

**Authors:** Brennen Heames, Filip Buchel, Margaux Aubel, Vyacheslav Tretyachenko, Dmitry Loginov, Petr Novák, Andreas Lange, Erich Bornberg-Bauer, Klára Hlouchová

**Affiliations:** 1 Institute for Evolution and Biodiversity, University of Münster, Münster, Germany; 2 Department of Cell Biology, Charles University, BIOCEV, Prague, Czech Republic; 3 Department of Biochemistry, Charles University, Prague, Czech Republic; 4 Institute of Microbiology, Czech Academy of Sciences, Prague, Czech Republic; 5 Department of Protein Evolution, MPI for Developmental Biology, Tübingen, Germany; 6 Institute of Organic Chemistry and Biochemistry, Czech Academy of Sciences, Prague, Czech Republic

**Keywords:** Molecular evolution, Origin of life, High-throughput screening

## Abstract

De novo gene emergence provides a route for new proteins to be formed from previously non-coding DNA. Proteins born in this way are considered random sequences and typically assumed to lack defined structure. While it remains unclear how likely a de novo protein is to assume a soluble and stable tertiary structure, intersecting evidence from random sequence and de novo-designed proteins suggests that native-like biophysical properties are abundant in sequence space. Taking putative de novo proteins identified in human and fly, we experimentally characterize a library of these sequences to assess their solubility and structure propensity. We compare this library to a set of synthetic random proteins with no evolutionary history. Bioinformatic prediction suggests that de novo proteins may have remarkably similar distributions of biophysical properties to unevolved random sequences of a given length and amino acid composition. However, upon expression in vitro, de novo proteins exhibit moderately higher solubility which is further induced by the DnaK chaperone system. We suggest that while synthetic random sequences are a useful proxy for de novo proteins in terms of structure propensity, de novo proteins may be better integrated in the cellular system than random expectation, given their higher solubility.

## Abstract (web summary)

While a bioinformatic comparison of de novo genes and synthetic random sequence does not identify a difference in biophysical properties, in vitro expression shows higher solubility for the de novo proteins, suggesting better integration into the cellular system than random sequences.

## Main

De novo genes, formed from previously non-coding DNA, have in recent years been confirmed as a ubiquitous feature of eukaryotic genomes and are likely to represent an important source of new protein-coding evolutionary material^1–3^. Translation of DNA that has not been under selection for its protein-coding capacity means that protein-coding de novo genes lie at the edge of yet-to-be-explored ‘dark protein space’^4^. Despite the unevolved nature of de novo-emerged proteins (here referred to as de novo proteins), many have been shown to play important functional roles. Examples include three mouse-specific de novo proteins with diverse cellular roles^5^, the yeast protein Bsc4, required for DNA-damage repair^6^ and codfish antifreeze glycoprotein^7^. We note that the genomic origin of some examples is not clear-cut and they are in this case referred to as ‘putative’ de novo; for confirmation of de novo origin, their ancestral sequences should be inferred as non-coding, as demonstrated in several recent studies^3,8^. Examples of new proteins for which ancestral non-coding sequences have not been confirmed but which are hypothesized to be de novo, include Goddard, Atlas and Saturn, which play essential roles in fly^9–11^.

Of the examples above, Goddard and Bsc4 have been structurally characterized and found to have maintained structural elements. However, both proteins appear to contain segments with high intrinsic disorder (ID). Others^6^ concluded that Bsc4 is best described as having a molten globule structure, suggesting that it may lack the defined folding funnel typical of many stable native folds.

Despite these examples, the structural properties of de novo proteins remain experimentally understudied. Computational prediction of the ID and aggregation propensity of de novo proteins has sparked hypotheses regarding the evolutionary pressure acting on newly emerged proteins^12–15^. Foremost is the suggestion that avoidance of aggregation is a critical selection pressure acting on new proteins^16^. Selection against aggregation would also explain why many studies identify higher ID in de novo proteins, given the fundamental link between amino acid hydropathy and ID^17^. More complete answers to these questions will come from experimental characterization, which should reveal the true distribution of aggregation propensity/ID in newly emerged protein sequences. Ultimately, systematic experimental characterization of new sequences should indicate if new proteins have the capacity to form folded structures and how frequently this occurs.

De novo proteins have sometimes been approximated to ‘random’ sequences on the basis of the lack of selection upon their emergence. However, de novo proteins emerge from existing genomes that are already known to carry different sequence and compositional biases, for example in GC content^18^. Diverse areas of research have shown that compositional biases can substantially impact protein properties such as translation efficiency, aggregation propensity and even specific attributes of ID^16,19,20^. The extent to which de novo and random sequences can be regarded as proxies therefore remains unclear. Moreover, random sequences represent true occupants of ‘dark protein space’^21^, whose properties themselves are heavily understudied. This region of sequence space has typically been assumed to contain non-functional and disordered proteins which are likely to be toxic and degraded if expressed in cells^22,23^.

Nevertheless, many recent studies have identified both structure and function in random proteins. Structure itself appears to be abundant in protein sequence space. Secondary structure occurrence has been reported to be remarkably close to that of biological proteins. In addition, 20–40% of random-sequence space has been observed to be resistant to proteolysis, probably due to tertiary structure formation^21,24–27^. Furthermore, we were recently able to demonstrate that while structured random proteins are hard to express in vivo due to their higher aggregation propensity, random proteins with greater ID are readily tolerated by *Escherichia coli*^26^. Simultaneously, at least some protein folds appear to be relatively evolvable from random sequences^28^. For example, Hayashi et al.^29^ were able to evolve an arbitrary random sequence to replace the D2 domain of an essential bacteriophage protein. Function through binding may be the most likely role that an unevolved protein could attain. For example, ATP-binders have been selected from pools of random proteins^30^. Random and partially randomized peptides have also been shown to have functional effects when expressed both in vitro and in vivo^31–35^. Finally, a smaller number of studies have evolved catalytic activity from randomized sequences, including esterase, barnase and RNA-ligase activity, the presence of which is itself an indicator of structured catalytic centres^36–39^. Altogether, while the above-listed studies suggest that both random and de novo proteins have non-zero structural and functional potential, their mutual relevance remains unclear.

Here, we set out to go further than previous studies by analysing the structural potential of putative de novo proteins. In doing so, we bring two strands of research together and experimentally characterize sets of (1) 1,800 putative de novo proteins identified in human and fly genomes and (2) 1,800 synthetically generated random sequences. While earlier studies were entirely computational or experimentally characterized single proteins, we quantify the properties of putative de novo proteins and compare them to ‘true’ random sequences, that is, unevolved and synthetically generated ones. We investigate two fundamental properties—solubility and structure content—using techniques previously unapplied to bulk analysis of putative de novo proteins.

We find that putative de novo proteins appear broadly similar to random sequences when length and amino acid frequencies are held constant. Consistent with computational prediction, the set of 1,800 putative de novo proteins we study had similar overall protease resistance to the set of synthetic random sequences. This indicates that, at least given the amino acid composition of the de novo sequences chosen, random sequences have similar structural potential. However, we also find that de novo proteins are (moderately) more soluble at this composition and structure level. This is indicative of some selective pressure having acted over the course of their real—albeit short—evolutionary histories.

## Results

### Library-based approach for investigation of de novo proteins

**Fig. 1:**
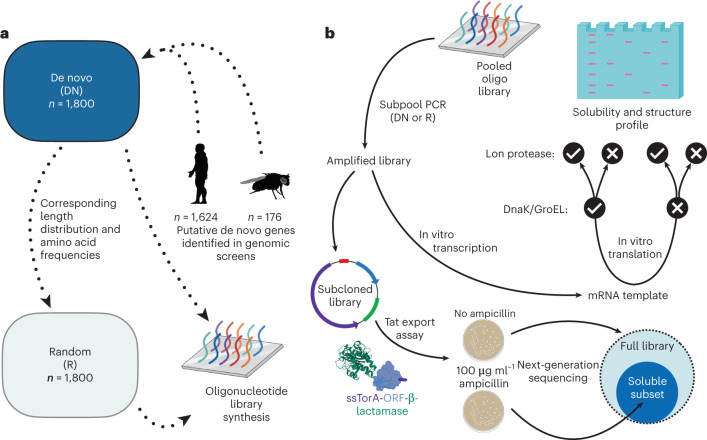
Library design, synthesis and experimental outline. **a**, Schematic illustration of the in silico design of libraries of de novo and unevolved random-sequence proteins. A de novo library (DN) was built from putative de novo proteins identified in human and fly. Subsequently, a library of unevolved random sequences (R) was designed to mirror the length and amino acid frequencies of library DN. The two libraries were synthesized by oligonucleotide library synthesis ready for experimental study. **b**, Approaches used to profile solubility and structure content of each library. Following amplification, each library was expressed in a chaperone-assisted cell-free format and structural content was quantified using a proteolytic assay. In parallel, subcloned libraries were expressed in *E. coli* to screen for soluble and folded variants that did not disrupt periplasmic export. Created with BioRender.com.

In this study, we combine computational and experimental characterization of two libraries: (1) a set of 1,800 putative de novo proteins identified in human or fly and (2) a set of 1,800 synthetic random sequences with no evolutionary history. Libraries were synthesized as an oligonucleotide pool, limiting proteins to 66 residues or less. A lower bound of 44 residues was chosen given the diminishing likelihood of domain-like structures for very short proteins. With these constraints, 1,800 sequences were selected from published sets of putative de novo proteins (Fig. 1a). Fly sequences (*n* = 176) are estimated to have emerged from previously non-coding intronic or intergenic regions less than 50 million years ago (Ma) and all are annotated as protein-coding genes in *Drosophila melanogaster* (151 of 176 fly sequences species-specific). Human sequences (*n* = 1,624) are unannotated intronic or intergenic open reading frames (ORFs) with *Homo sapiens*-specific expression (born <6.7 Ma). We refer to the fly and human subsets of library DN as ‘putative de novo proteins’. In both cases, proteins were found to have weak, tissue-specific expression and low-to-moderate signals of selection. As a further assessment of the human-specific sequences, we examined conservation across genomes from four human populations which indicated that these proteins are mostly fixed rather than segregating (Supplementary Fig. 12).

Given the recent acquisition of these proteins and their apparent unevolved nature, it remains unclear how these new proteins differ from ‘true’ random sequences if at all. For both human and fly sequences, various protein properties were predicted. Fly de novo proteins were compared to randomly sampled intergenic sequences without expression evidence and found to have higher GC content and ID. Human-specific ORFs identified by Dowling et al.^14^, which make up most of the library DN, were not compared to a ‘more random’ set of sequences. However, they were found to have lower GC content than conserved ORFs (‘conservation level 5’, with exon overlap) but similar predicted ID. This discrepancy between GC content and ID may be explained by the action of selection, either on newly emerged proteins towards high ID or over longer evolutionary timescales to shape the properties of highly conserved ORFs towards lower GC content while keeping ID constant.

To identify such selection towards a given biophysical property, a natural and feasible approach is to compare the set of putative de novo proteins to ‘true’ random controls and see if they differ. For this reason, a synthetic random library (R) was designed, with amino acid frequency and length distributions matched to library DN. Given that amino acid composition is a major determinant of all biophysical properties, the specification of library R should provide the most appropriate comparison; any differences in protein property between DN and R should be attributable to the specific residue ordering (and not overall compositional bias; Supplementary Fig. 3). We note that this experimental design does not make our synthetic random library true unevolved precursors, given that they were not taken from a genomic sequence. For this reason, sequence biases other than amino acid composition may make them differ compared to our putative de novo library.

### Sequence-based prediction of biophysical properties

**Fig. 2:**
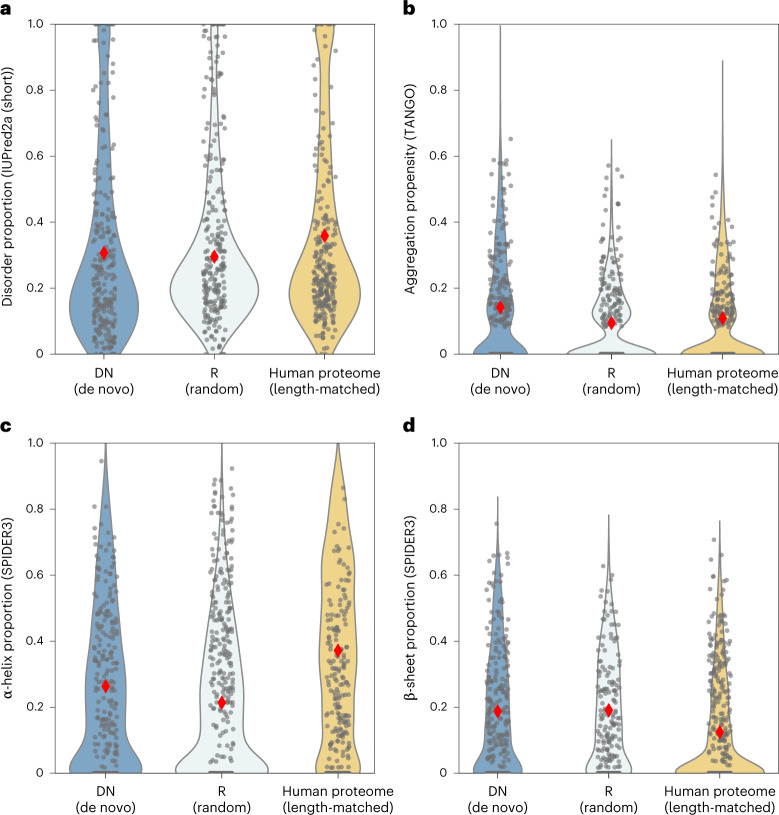
Biophysical predictions are similar for de novo and unevolved random sequences and suggest that both harbour high structural potential. **a**–**d**, Libraries DN (dark blue) and R (pale blue), designed to have matched length and amino acid frequencies, are predicted to have highly similar biophysical properties as expected. Comparison to a length-matched subset of the human proteome (yellow) shows broadly similar predictions (ID propensity (**a**); aggregation propensity (**b**); α-helix proportion (**c**); β-sheet proportion (**d**)), suggesting that native-like properties are present in or at least evolutionarily accessible to, random-sequence proteins. Red diamonds indicate mean value of distributions, which are subsampled to 250 sequences for visualization.

Having designed libraries of putative de novo (library DN) and synthetic random proteins (library R) in silico, we next made some bioinformatic predictions of their protein properties. Figure 2 shows predictions for four fundamental features. To put biophysical properties in context with those of conserved (native-like) proteins, predictions are compared to a length-matched subset of 3,600 annotated human proteins. In all cases, predictions for DN and R are highly similar. Predictions of ID distribute similarly for all three classes (Fig. 2a), as does aggregation propensity (Fig. 2b). Comparison to annotated human proteins suggests reduced propensity for α-helices in both libraries (Fig. 2c) but higher propensity for β-sheets (Fig. 2d). Accordingly, from primary sequence alone, libraries DN and R appear to have appropriate levels of hydrophobic and hydrophilic residues to form native-like structural content.

Further sequence properties are shown in Supplementary Fig. 1; in addition, in Supplementary Fig. 2 we show sequence properties split by species. This identifies that our fly-based libraries have higher predicted ID than human-based libraries, as expected given the relatively high GC-contents of drosophilid genomes. Aside from predicted biophysical properties, we also looked for differences in sequence information content that could result from the random amino acid sampling used to generate library R. We find that overall sequence information content is highly comparable for DN, R and conserved proteins (Supplementary Fig. 5a); however, library R is depleted in short low-complexity regions compared to DN (Supplementary Fig. 5b).

Prediction tools such as IUPred have been trained using the (relatively small) sets of proteins for which disorder or aggregation has been determined experimentally. Given the new and unevolved nature of our libraries, we looked for a more generalizable predictor of structural content or stability. Learned embeddings have been described recently as a way to encode fundamental protein features learned over much larger regions of sequence space than have been experimentally characterized^40^. For example, using UniRep embeddings as input, a linear model was shown to outperform Rosetta total energy predictions when trained on protease sensitivity data^41,42^.

Before an experimental protease assay (see following sections), we implemented this predictive model to generate protease stability scores for each library. As shown in Supplementary Fig. 4, we find libraries DN and R have highly similar predictions. The control set of annotated human proteins are predicted to be marginally more stable on average. However, scores broadly overlapped with those for the DN and R. The stability values predicted here are expected to correlate with total structure content and globularity. Accordingly, together with secondary structure predictions (Fig. 2), both libraries appear to have potential for structural content similar to that of conserved proteins. While de novo proteins may distribute to a particular region of protein sequence space—either due to selection or as a byproduct of their occurrence in a genome—library R is not similarly constrained. Instead, the similarity of all predictions for DN and R with those for conserved proteins appear to result from their similar amino acid compositions.

Aside from illustrating that all random sequences with appropriate amino acid composition may have structure-forming potential, the predictions made here demonstrate that any structural differences between this set of putative de novo proteins and their unevolved random counterparts are indistinguishable computationally. This hypothesis is entirely plausible but testing it computationally relies on the accuracy of the predictors used; predictors which may not be sensitive to small differences, especially when compositional biases are removed. For this reason, we next sought to validate these predictions experimentally.

### A cell-based export assay identifies soluble library members

Following in silico design, the libraries DN and R were synthesized as an oligonucleotide pool (Fig. 1a). De novo and random subpools were PCR amplified from this pool and used as a starting point for subsequent experimental work. We first used a twin-arginine export quality assay, which relies on translocation of β-lactamase via the twin-arginine translocation (Tat) pathway, to screen for soluble members of each library^43^. This assay is implemented by subcloning each library to a vector encoding an N-terminal secretion signal and a C-terminal β-lactamase (construct illustrated in Fig. 1b). Upon expression of the resulting fusion constructs in *E. coli*, successful export of the fused β-lactamase can be detected by colony formation on ampicillin plates. Ampicillin can therefore be used to select for library members that do not interfere with translocation. Twin-arginine export assay was previously shown to select for soluble target protein^44^ and remove gene synthesis errors^45^. We here use the assay to select for (and subsequently identify by sequencing) the soluble subsets of each library that do not result in aggregation of β-lactamase fusion proteins.

**Fig. 3:**
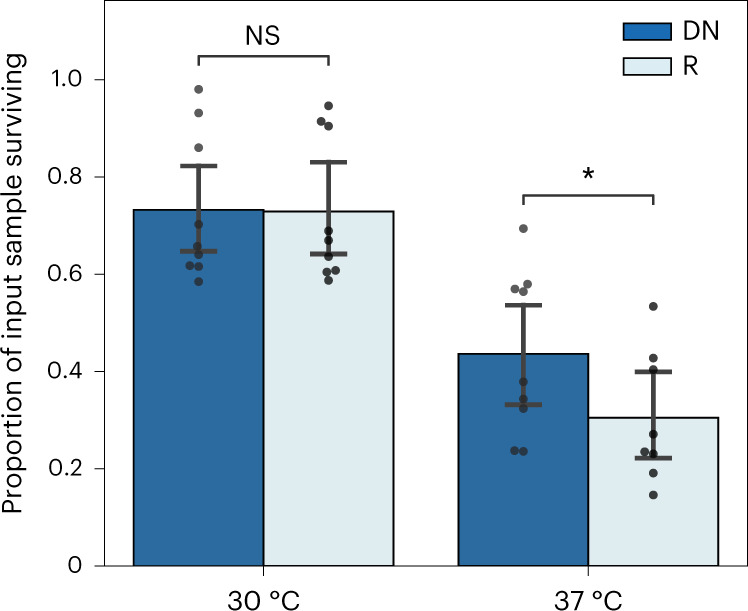
A cell-based assay identifies subsets of each library with potential for soluble expression. NGS of input (plated without ampicillin) and selected libraries (+100 μg ml^−1^ of ampicillin) allows quantification of changes in library diversity following twin-arginine export assay. The proportion of the input sample surviving selection on ampicillin is shown for libraries DN and R at 30 °C and 37 °C. Survival at 30 °C was very similar for both libraries (71.3% versus 69.7%), while at 37 °C library DN has significantly higher survival than library R (43.6% versus 30.5%) (one-tailed *t*-test, unadjusted *P* = 4.9 × 10^−2^; error bars show 95% confidence intervals around the mean; number of biologically independent samples following outlier exclusion: *n* = 9, 9, 9, 8). NS, not significant.

Selection of libraries DN and R on ampicillin, followed by NGS-based quantification of library diversity (the number of unique sequences represented), allows identification of soluble subsets of each library (and additionally an assessment of library quality; Supplementary Table 1). When plated without ampicillin at 30 °C over three-quarters of theoretical library diversity (the number of sequences synthesized for the library) was identified above a threshold of 100 reads-per-million (DN 76.6% (±4.3%), *n* = 1,800; R 81.4% (±3.2%), *n* = 1,800; for read-count distributions see Supplementary Fig. 7). Post-selection on 100 μg ml^−1^ of ampicillin, the fraction of the library identified by sequencing dropped to 54.1% (±9.5%) and 56.3% (±11.9%) for libraries DN and R, respectively. The proportion of input library surviving selection is shown in Fig. 3. This indicates that both libraries are moderately soluble when expressed as β-lactamase fusions in *E. coli*, with no difference between libraries DN and R at 30 °C.

Repeating the same assay at 37 °C (Fig. 3), we found similar diversity on preselection plates (DN 75.6% (±4.5%), *n* = 1,800; R 77.6% (±5.1%), *n* = 1,800). However, a greater drop in representation was seen upon ampicillin selection to 32.7% (±12.2%) and 26.4% (±11.9%) for libraries DN and R, respectively. A greater efficacy of selection for solubility at 37 °C is consistent with greater overexpression than at 30 °C—and could also indicate the presence of slow folders which are less able to avoid aggregation at increased temperatures. Aggregation of de novo proteins expressed recombinantly has been noted previously and is consistent with this result^10^. As shown in Fig. 3, a greater proportion of library DN survives ampicillin selection compared to library R when assayed at 37 °C. Survival can additionally be broken down by species (Supplementary Fig. 8), showing consistent trends for both human and fly subsets.

### Probing intrinsic library solubility in a cell-free system

**Fig. 4:**
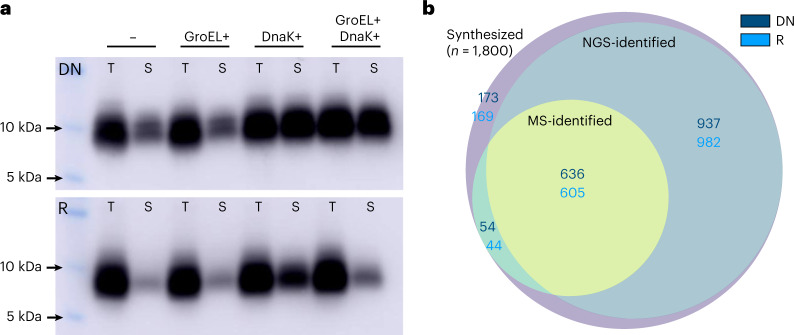
Cell-free expression shows putative de novo proteins to be more soluble than synthetic random sequences. **a**, Western blot showing total (T) and soluble (S) fractions of bulk library expression using reconstituted *E. coli* machinery in cell-free format at 37 °C. Library DN (top row) is marginally more soluble than library R (bottom row). Cotranslational chaperone addition (DnaK, GroEL or both) shows that GroEL has little effect but that DnaK solubilizes both libraries equally well. **b**, To check that cell-free expression results in similar protein synthesis of a large fraction of libraries DN and R, mass spectrometry (MS) was used to quantify protein-level diversity of the synthesis reaction in the presence of cotranslational DnaK. Over a third of each library (690 and 649 proteins for DN and R, respectively) were identified by MS, putting a lower bound on the diversity of protein expression. Overlap with NGS-identified sequences from the twin-arginine export assay is also shown. Source data

To further investigate the properties of our putative de novo and true random sets, libraries were expressed in a cell-free format using a reconstituted *E. coli* expression system including transcriptional and translational machinery. Cell-free (in vitro) recombinant expression has two key benefits in this case: first, it allows tight control of expression conditions and control of cofactor concentrations; and, second, it separates intrinsic target-protein behaviour (for example, aggregation propensity) from the complex cellular milieu^46^. Libraries were expressed in vitro with a C-terminal FLAG-tag and target protein detected by western blot (Fig. 4a). In addition to total yield (T), the subset of soluble library protein is isolated and loaded in adjacent lanes (S). The ratio of intensities of the ‘soluble’ and ‘total’ lanes therefore provides an estimate of the fraction of soluble expression in each sample.

Base expression (Fig. 4a) was compared to yield in the presence of molecular chaperone systems added to the cell-free reaction (Methods). GroEL/ES and DnaK systems were added cotranslationally, that is were present from the start of the reaction. As can be seen in Fig. 4a, soluble protein makes up only a fraction of total expression in the absence of DnaK. This was true for both the putative de novo proteins (top row) and the random sequences (bottom row). The same trend for DN to be moderately more soluble than R is seen here, as with the twin-arginine assay at 37 °C. We also observe a slightly higher band for basal soluble expression (versus total expression). However, given that gel migration is not fully quantitative with respect to molecular weight, we do not speculate here about the molecular weight distribution of soluble and total expression^47^.

Upon addition of GroEL/ES system (GroEL+), no major difference in soluble yield was seen for either library. However, upon DnaK addition (DnaK+) both libraries were highly solubilized (seen by intensity in lane S being close to that in lane T). When both DnaK and GroEL/ES systems were added, the improved solubility was maintained for library DN. However, for library R, addition of GroEL/ES appeared to counteract the effect of DnaK and solubility dropped closer to basal levels. A possible explanation for this is unproductive interaction of GroEL with the synthetic library R sequences impeding the action of DnaK. While the random proteins are being refolded inside the GroEL complex unsuccessfully, DnaK would be unable to bind and perform its function. A similar trend of decreased protein expression upon chaperone addition was observed by Eicholt et al.^48^ for expression of de novo proteins.

Supplementary Fig. 6 shows predicted DnaK binding sites for each library, compared to the set of length-matched annotated human proteins. Library sequences are predicted to have on average four regions for which DnaK should have high affinity (short hydrophobic regions with positively charged residues). This is comparable to the prediction for conserved proteins, which may help explain why DnaK is effective and acts similarly for libraries DN and R (giving about threefold solubility increase).

To verify that cell-free expression resulted in a high proportion of the synthesized libraries being translated, mass spectrometry (MS) was used to identify tryptic peptides following FLAG-based purification. Over a third of libraries DN and R were identified by MS following expression at 37 °C in the presence of DnaK. As shown in Fig. 4b, most sequences identified by MS were also identified in preselection NGS reads at the same temperature in the twin-arginine export assay (Fig. 3; across the three replicate NGS samples). Although NGS and MS data are based on cellular and cell-free expression, respectively, and in different constructs, we also see a signal for MS-identified sequences to have higher NGS read counts (Supplementary Fig. 11a), suggesting that the remaining sequences not identified by MS may be below the detection threshold. Finally, the highly similar distributions of peptide intensities for libraries DN and R (Supplementary Fig. 11b) points to comparable expression levels across both libraries.

### Proteolytic assay identifies undegradable library subsets

We next investigated the structural content using a Lon-based proteolytic assay^27,49^. Using the same cell-free expression system (Fig. 4a), Lon protease was added to reaction mixtures. The preference of Lon for non-specific cleavage of exposed hydrophobic regions means that it causes the greatest amount of degradation for IDP-like proteins and in general for proteins with lower structural propensity.

**Fig. 5:**
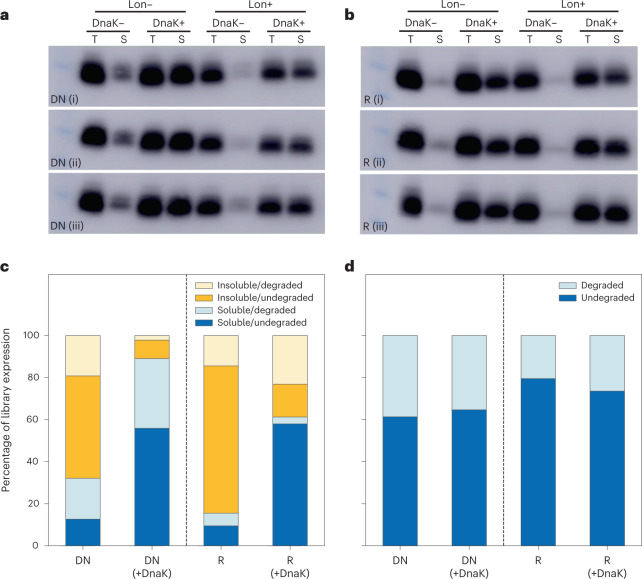
Quantification of degraded library fractions following cell-free expression in the presence of Lon protease. **a**,**b**, Total (T) and soluble (S) expression with cotranslational addition of DnaK and/or Lon protease at 37 °C: triplicate western blots shown for libraries DN (**a**) and R (**b**). Non-specific cleavage of hydrophobic regions by Lon protease results in preferential degradation of disordered proteins, with a visible net reduction in yield for Lon+ samples. **c**, Quantification of degraded fractions with respect to solubility reveals a greater IDP-like (soluble/degraded) fraction for putative de novo proteins versus ‘true’ random sequences. DnaK addition, however, results in a greater increase in the soluble/undegraded fraction than the IDP-like fraction (for both DN and R). **d**, Summary of degraded versus undegraded fractions, regardless of solubility (sum of dark and light bars in **c**, respectively). Library R is marginally less degradable than DN, suggesting slightly higher structural propensity (one-tailed *t*-test, R/R + DnaK versus DN/DN + DnaK, *P* = 1.67 × 10^−2^). Source data

Figure 5a,b show triplicate blots for libraries DN and R, respectively, with addition of DnaK and Lon protease to cell-free reaction mixtures. Quantification of blot intensity over replicate blots allows an estimation of the degradable fractions of each library with respect to solubility (Methods). This is illustrated in Fig. 5c, with soluble fractions (blue hues) split by degradability (dark blue, soluble/undegraded; pale blue, soluble/degraded). The degraded and undegraded fractions of insoluble yield can also be inferred in this way (dark yellow, insoluble/undegraded; pale yellow, insoluble/degraded). Quantification in all cases supports our main finding that library DN has higher intrinsic and chaperone-supported solubility compared to library R (one-tailed *t*-test; *P* = 1.48 × 10^–3^ (no chaperone), *P* = 6.30 × 10^–4^ (DnaK+); Supplementary Fig. 10).

As can be seen in Fig. 5a,b, addition of Lon protease causes a reduction in both the total yield and that of the soluble subset (where degradation is most visible). The fact that some soluble protein remains undegraded points to a degree of structural content even for the soluble fraction. In other words, a fraction of both the de novo and true random proteins has soluble expression, not all of which consists of IDP-like proteins (soluble and disordered). Quantifying this in Fig. 5c shows that, considering only the soluble fraction, library DN has a greater proportion of these IDP-like proteins than library R, where less of the soluble fraction was degraded than not. In the insoluble fraction, for both libraries most protein is inferred as undegradable. We suggest that this corresponds to insoluble proteins with above-average structural potential.

With addition of the DnaK, the same solubility increase as before (Fig. 4a) was seen. Comparing library DN to its no-DnaK reference suggests that DnaK has acted to prevent much of the soluble/undegraded fraction from converting to the insoluble/undegraded fraction. Similarly, DnaK appears to have prevented much of the soluble/undegradable fraction of library R from aggregating. However, solubilization of library R does not appear to result in a concurrent increase in the soluble/degraded fraction (IDP-like). This may be best explained by the overall lower degradation seen for library R. Combining soluble and insoluble fractions, library R can be seen to have higher apparent structural propensity compared to library DN (Fig. 5d).

## Discussion

Given an emerging picture of abundant structure and function within sequence space, an outstanding question is if de novo proteins differ from other classes of random protein. In other words: do de novo proteins occupy a privileged area of sequence space with respect to structure or function? Direct attempts to answer this question have so far not been made. Instead, experimental evidence from unnatural random-sequence libraries have formed the basis for many hypotheses regarding de novo emergence. Further, direct investigation of de novo proteins has been limited to either computational prediction or experimental characterization of individual proteins. Going beyond these studies, we assess a library of putative de novo proteins experimentally and compare their properties to a matched library of unevolved random sequences. In doing so, we show that recently emerged putative de novo proteins behave similarly to unevolved counterparts but that the set of putative de novo proteins harbours a larger fraction of soluble and protease-sensitive sequences.

Recent improvements in DNA synthesis technology have made it feasible to generate large libraries of high-fidelity sequences. Using oligonucleotide library synthesis, it is possible to investigate proteins in high-throughput by direct specification of their coding sequences. We focus on short de novo proteins (<66 amino acids) that we previously identified in human and fly, which can be synthesized directly in a single oligonucleotide. However, multiplex gene synthesis also makes this approach applicable to longer proteins specified over multiple oligos^45,50^. Libraries generated in this way should ultimately allow coupling of computational identification and high-throughput investigation of diverse protein sequences.

Having designed a library of 1,800 random sequences (R) to have matched amino acid frequencies and lengths as a set of 1,800 putative de novo sequences (DN), we ran primary sequence-based predictions for several biophysical properties. Given that all computational predictions are highly similar between the two libraries, a possible conclusion is that our library of de novo proteins is generally close to the set of synthetic random sequences and that their shared biophysical propensities result from their matched amino acid compositions. However, the reliability of predictions for random-type proteins remains ambiguous, given that it is only possible to validate prediction tools on well-characterized proteins which are typically well conserved. Furthermore, the predictors rely heavily on sliding-window assessments of sequence composition which could struggle to differentiate DN and R. In light of this, experimental characterization remains critical to any conclusions regarding this class of proteins; a step that has until now not been reported for more than a handful of de novo proteins.

We first assessed solubility of our libraries using a twin-arginine export quality assay^43^, shown to select for soluble and folded proteins^45^. Sequencing of libraries DN and R after selection showed that at least two-thirds of each library (71.3% and 69.7%, respectively) has potential for soluble expression at 30 °C. Interestingly, computationally predicted properties did not correlate with those sequences most enriched by selection (the most soluble variants). Any distinguishing properties of these sequences were therefore not captured by computational tools, further highlighting the need for experimental characterization.

Next, we expressed each library in cell-free format using reconstituted *E. coli* expression apparatus. Given that the putative de novo proteins were sourced from human and fly, cell-free expression allows separation of the inherent biophysical properties of each library and the unnatural *E. coli* cellular environment. In addition, the cell-free format enables systematic changes to expression conditions—including addition of molecular chaperones to aid solubility or proteases to assess protein stability. In the absence of chaperones, we found putative de novo proteins to have significantly higher solubility than their unevolved random counterparts (~30% soluble versus ~15%). This trend is in agreement with the twin-arginine export assay, with a larger fraction of the de novo library having soluble potential at 37 °C. The higher solubility of putative de novo proteins may reflect their exposure to selection; avoidance of aggregation has been suggested as a key selective pressure on new proteins^16^. Despite their recent emergence, and typically low and tissue-specific expression, selection may have shaped the properties of these sequences to some degree.

We next tested the effect of two chaperone systems, GroEL and DnaK, on the expression of each library. While GroEL had no effect on solubility or overall expression, DnaK increased the soluble fraction of both libraries by around threefold. This resulted in soluble fractions of ~90% (DN) and ~60% (R), probably due to DnaK having similar effectiveness on both libraries and preventing approximately equal amounts of protein from forming insoluble aggregates. The effectiveness of DnaK on random proteins was demonstrated recently^27^. Confirming this result for putative de novo proteins indicates that DnaK (or its eukaryotic homologue Hsp70) may be essential for avoidance of aggregation in the early stages of protein evolution.

Finally, to probe the structural content of each library, we included Lon protease in the cell-free expression system^49^. By preferentially cleaving exposed hydrophobic regions of unstructured proteins, Lon degradation correlates with ID^51^. A Lon-based method was recently used to probe random-sequence libraries of different amino acid compositions^27^, identifying a substantial proportion of the soluble fraction of each library to be resistant to degradation. In addition, increasing solubility with DnaK also had a small effect on the fraction of non-degradable protein. While the precise fractions of degraded protein for each condition should be interpreted with care, in both cases over 50% of soluble protein was not degraded by Lon upon DnaK addition. A subset of each library may therefore harbour structural elements that interfere with cleavage, in agreement with findings that structure is abundant in sequence space^27^. However, the low resolution of the Lon-assay prevents differentiation of different forms of structural elements, such as oligomeric or molten globule. Interestingly, we find 10–20% higher degradation for putative de novo proteins compared to synthetic random sequences, in agreement with our earlier report showing that unevolved sequences with less structural content are more soluble upon expression in *E. coli*^26^.

Although putative de novo proteins appear marginally more soluble than synthetic random proteins, both show sensitivity to molecular chaperones. Similarly, while a subset of both libraries may harbour structural content, putative de novo proteins appear to contain more disordered regions, in correlation with their higher solubility. We note that our study is limited to short proteins of a specific composition and GC content distribution. While the results presented here transcend earlier computational analyses and studies of single de novo proteins, we note that it is highly challenging to prove ultimately any instance of de novo emergence and there remains a degree of uncertainty about the true origin of the putative de novo proteins studied here. Some of the putative de novo set, in particular those from *H. sapiens*, may be transient short-lived protogenes which have not yet assumed critical cellular roles (but are nonetheless evolutionarily highly relevant^52^).

In summary, we suggest that de novo proteins of the sort studied here are not especially privileged among random sequences and that the propensity for structure across sequence space may be key to the feasibility of de novo emergence. However, our findings of higher solubility for putative de novo proteins are consistent with early selection pressure to avoid aggregation. To corroborate this finding, larger numbers of de novo proteins drawn from diverse genomic backgrounds and conservation levels should be characterized in future efforts.

## Methods

### Library sequence selection

To study the properties of de novo and random-sequence proteins experimentally, two libraries were first designed in silico. In prior work, we identified large sets of putative de novo proteins which appear to have emerged from previously non-coding DNA. To build a de novo library (DN), 1,800 proteins were selected from two studies identifying de novo genes in fly (*n* = 176)^15^ and newly transcribed human ORFs (*n* = 1,624) (‘conservation level 0’ in Dowling et al.^14^, excluding ORFs with exon overlap). A library of 1,800 unevolved random-sequence proteins (R) was then generated synthetically by sampling amino acids using the frequency distribution of library DN. Sequence lengths were also matched to those of library DN, so that library R had identical length and amino acid composition to library DN.

### Oligonucleotide pool design

Libraries DN and R were synthesized as a SurePrint oligonucleotide pool by Agilent (DE). Oligonucleotides were specified to include NdeI and XhoI restriction sites 5ʹ and 3ʹ to the CDS for downstream cloning. Additionally, 15 base pair (bp) primer sites were added upstream and downstream of the restriction sites to allow libraries DN and R to be PCR amplified separately from the oligo pool. The DnaChisel package^53^ was used to codon optimize CDSs for protein expression in *E. coli*, while avoiding introduction of undesired restriction sites and homopolymer repeats of 5 bp or longer. Starting from desired amino acid sequences, we selected the highest frequency codon according to *E. coli* K12 frequencies (http://www.kazusa.or.jp/codon) and the ‘harmonized Relative Codon Adaptiveness’ implementation of DnaChisel was used to replace rare codons^54^. Code to generate optimized oligo pools was used here as follows to select and optimize the 1,800 longest compatible ORFs from a list of human and fly de novo ORFs:


python build_oligos.py -i denovo_orfs.csv -s e_coli -c harmonize_rca-t h_sapiens -n 1800 -r 1 -d primers.db -p 15 -fL CAT -fR CTCGAG


### Prediction of protein properties

Intrinsic structural disorder and globularity were calculated using IUPred2a (ref. ^55^); secondary structure, Phi and accessible surface area were predicted using SPIDER3 (ref. ^56^); aggregation propensity was predicted using TANGO^57^; isoelectric point (IEP) was predicted using EMBOSS pepstats^58^; and grand average of hydropathy (GRAVY) index was calculated using CodonW^59^. To predict stability scores, we used an implementation of UniRep^42,60^ to generate sequence embeddings of size 1,900 and trained a sparse linear model (Lasso least-angle regression with tenfold cross-validation) on a dataset of de novo-designed proteins with experimentally determined stability scores^41^, as described by Alley et al.^42^. As a comparison for predictions, 3,600 annotated human proteins (Ensembl 97 *H. sapiens* proteome) were selected by random sampling of an equal-length protein for each member of library DN. DnaK binding sites were predicted using the ChaperISM suite (v.1) in quantitative mode with default settings^61^. Amino acid repeat content was calculated using the fLPS package^62^.

### Twin-arginine export quality assay

To screen for soluble proteins, libraries were expressed as fusions with an N-terminal Tat secretion signal (ssTorA) and a C-terminal β-lactamase. Misfolding or aggregation of the target ORF should prevent secretion of the construct to the *E. coli* periplasm, allowing selection by plating on increasing concentrations of ampicillin. Libraries DN and R were PCR amplified separately from the oligonucleotide pool, with primers introducing EcoRI and BamHI restriction sites. After restriction cloning to pSALECT-EcoBam (Addgene plasmid 59705), libraries were transformed by electroporation to *E. cloni* 10G (Lucigen, 60106-1) in triplicate, with each transformation plated three times for a total of nine replicates. Whole transformations were plated on LB agar + 25 μg ml^−1^ of chloramphenicol and grown overnight. Libraries were then scraped from plate into LB medium adjusted to have the same optical density OD_600_. The assay involved plating equal volumes on LB agar supplemented with either: 25 μg ml^−1^ of chloramphenicol or 25 μg ml^−1^ of chloramphenicol and 100 μg ml^−1^ of ampicillin. After incubation overnight at 30 °C, plates were scraped into PBS and plasmid isolated (GeneJET Plasmid Miniprep Kit, Thermo Scientific, K0502). Primers encoding 8 bp 5′ and 3′ barcodes were used to amplify samples from each condition (Supplementary Table 2).

### Next-generation sequencing

Amplicons from twin-arginine export assay conditions were purified, combined in equimolar amounts and amplicon size distribution (270–350 bp) verified by capillary electrophoresis. Amplicons were subsequently sequenced using an Illumina MiSeq platform. Reads were merged, trimmed and filtered to remove low-quality reads using the fastp suite^63^. The cutadapt suite^64^ was used for read demultiplexing and reads were then mapped to CDS sequences of libraries DN and R using the Burrows–Wheeler alignment MEM algorithm^65^. SAMtools was used for conversion to SAM file format, sorting and indexing^66^. Finally, reads mapped to each variant were counted using HTSeq^67^. Read counts were converted to reads-per-million reads values (per plating condition) to control for sequencing depth and sequences were subsequently filtered using a threshold of 100 reads-per-million to remove those with very low abundance (<0.01% of reads in a given sample).

### Cell-free expression and Lon proteolytic assay

Both protein libraries were produced in a cell-free expression system to evaluate their solubility, response to chaperones and structural content (using proteolysis resistance) in a cell-like environment. Expression from messenger RNA templates was carried out in *E. coli* reconstituted cell-free system and solubility was assessed by centrifugation to separate soluble fraction, followed by quantitative western blot. Bacterial Lon protease preferentially cleaves unstructured proteins and was added to the reactions to investigate proteolytic resistance potential of the protein libraries^27,49,51^.

First, library subpools were PCR amplified to introduce EcoRI and BamHI restriction sites, subcloned into pET24a+ vector modified to encode a C-terminal FLAG-tag and electroporated into *E. cloni* 10G (Lucigen, 60106-1). Cells were grown overnight at 37 °C on LB agar + 50 μg ml^−1^ kanamycin plates and transformants scraped for plasmid DNA isolation. The region containing the T7 promoter, library sequence and terminator was PCR amplified to serve as template for in vitro transcription (NEB HiScribe T7 kit, E2040S). The PUREfrex 2.0 system (GeneFrontier Corporation, PF201-0.25-EX) was used for in vitro translation. The reactions were mixed as per protocol to final volume 10 μl with addition of 0.05% Triton X-100 and incubated at 37 °C for 2 h. To assess the effect of molecular chaperones on the soluble yield of protein expression, reactions were supplemented with DnaK or GroE mix (GeneFrontier Corporation, PF003-0.5-EX and PF004-0.5-EX), to final concentration of 5 μM DnaK, 1 μM DnaJ and GrpE, 0.1 μM GroEL and 0.2 μM GroES. For proteolytic resistance assay, purified Lon protease was added cotranslationally at 0.1 μM working concentration.

Following production all reactions were halted by adding 40 μl of puromycin buffer (300 μM puromycin, 50 mM Tris, 100 mM NaCl, 100 mM KCl, pH 7.5) and incubating at 30 °C for 30 min. Next, 5 μl of such mixture was processed for SDS–polyacrylamide gel electrophoresis serving as the total (T) fraction of expression, while the rest was centrifuged (21,000*g*, 30 min, 21 °C). Soluble (S) fraction was collected by taking 5 μl of the supernatant. Finally, three technical replicates for each sample were analysed by SDS–PAGE and western blot using Anti-FLAG (Sigma-Aldrich Monoclonal ANTI-FLAG M2-Peroxidase (HRP), A8592). Images were quantified using ImageJ (US National Institutes of Health).

### Mass spectrometry analysis of expressed proteins

Libraries DN and R were expressed in a cell-free system following the protocol described in the previous subsection. Reactions were scaled up to a final volume of 125 μl and supplemented with DnaK mix (5 μM DnaK, 1 μM DnaJ and GrpE) and 0.05% (v/v) Triton X-100. A total of 100 μl of ANTI-FLAG M2 Magnetic Beads (50 μl of packed gel; Sigma-Aldrich, M8823-1ML) was equilibrated four times with ten packed gel volumes of binding buffer (50 mM Tris, 150 mM NaCl, 0.05% (v/v) Triton X-100, pH 7.5). The samples were diluted tenfold with the binding buffer, centrifuged at 21,000*g* at 4 °C for 30 min, mixed with the beads in 1.5 ml centrifugation tubes and incubated for 1 h at room temperature on a tumbler. Following the binding, the beads were washed four times with 20 packed gel volumes of washing buffer (50 mM Tris, 150 mM NaCl, pH 7.5) and incubated with 500 μl of 0.5 M ammonium hydroxide for 20 min on a tumbler. Finally, eluted proteins were transferred to a fresh centrifugation tube and stored at −20 °C.

Samples collected after affinity purification were twice diluted with 100 mM 4-ethylmorpholine/acetate buffer (pH 8.5):acetonitrile (ACN) (90:10 v/v) followed by overnight trypsin digestion (protein:enzyme ratio, 1:20) at 37 °C. Digestion was stopped by addition of TFA to a final concentration of 0.1% and the resulting digest was subsequently dried by a SpeedVac (Eppendorf) to reach 30 μl of final volume. For each sample, 1 μl was analysed on an ultrahigh pressure nanoflow chromatography system (Vanquish Neo, Thermo Fisher Scientific) coupled to a trapped ion mobility quadrupole time-of-flight mass spectrometer (timsTOF Pro SCP, Bruker Daltonics) via a nanoelectrospray ion source (Captive Spray Source, Bruker Daltonics). Peptides were separated on an analytical column (25 cm × 75 μm, C18, 1.6 μm) (Dr. Maisch). Peptides were eluted using 2% ACN/0.1% formic acid as mobile phase A at a flow rate of 400 nl min^−1^ and 45 min-long gradient with liner increase of acetonitrile to 35% (the mobile phase B was ACN/0.1% formic acid) at a 50 °C column oven temperature. The eluting peptides were interrogated by an MS acquisition method recording spectra from 100 to 1,700 *m*/*z* and ion mobility scanned from 0.6 to 1.6 V s cm^−2^. The method consisted of a TIMS survey scan of 150 ms followed by six PASEF MS/MS scans, each 150 ms for ion accumulation and ramp time. The total cycle time was 1.08 s. Target intensity was 40,000, the intensity threshold was 1,000 and singly charged peptides with *m*/*z* < 800 were excluded by an inclusion/exclusion polygon filter applied within the ion mobility over *m*/*z* heatmaps. Precursors for data-dependent acquisition were fragmented with an ion mobility-dependent collision energy, which was linearly increased from 20 to 59 eV. Raw data were processed using Andromeda^68^ search engine integrated in MaxQuant environment v.1.6.17.0 (ref. ^69^). Experiment type was set as TIMS-DDA with default parameters. Data were searched against a custom-made database containing target sequences. Search parameters were used as follows: methionine oxidation was set as a variable modification; trypsin was set as enzyme with one missed cleavage (unspecific digestion was set as enzyme specificity), false discovery rate was set to 1%. The obtained results were further processed using Perseus v.2.0.7 (ref. ^70^).

### Reporting summary

Further information on research design is available in the Nature Portfolio Reporting Summary linked to this article.

## Supplementary information

Supplementary Information Supplementary Figs. 1–10 and Tables 1–4.

Reporting Summary

## Data Availability

Library sequences, twin-arginine assay sequencing reads and processed data files are deposited under Zenodo 10.5281/zenodo.7556935. Source data are provided with this paper.

## Source data

Source Data Fig. 4 Unprocessed western blots.
Source Data Fig. 5 Unprocessed western blots.

## Code availability

Code used for library design can be found at https://zivgitlab.uni-muenster.de/ag-ebb/de-novo/de_novo_lib.
