# Supplementary material for: Experimental characterization of de novo proteins and their unevolved random-sequence counterparts
Source: Nat Ecol Evol. 2023 Apr 6;7(4):570–80. doi: 10.1038/s41559-023-02010-2 (PMC10089919; doi:10.1038/s41559-023-02010-2)
Supplement: Supplementary file 1 — Supplementary Figs. 1–10 and Tables 1–4. [file 41559_2023_2010_MOESM1_ESM.pdf]

nature ecology & evolution

Article

https://doi.org/10.1038/s41559-023-02010-2

Experimental characterization of de novo proteins and their unevolved random-sequence counterparts

In the format provided by the authors and unedited

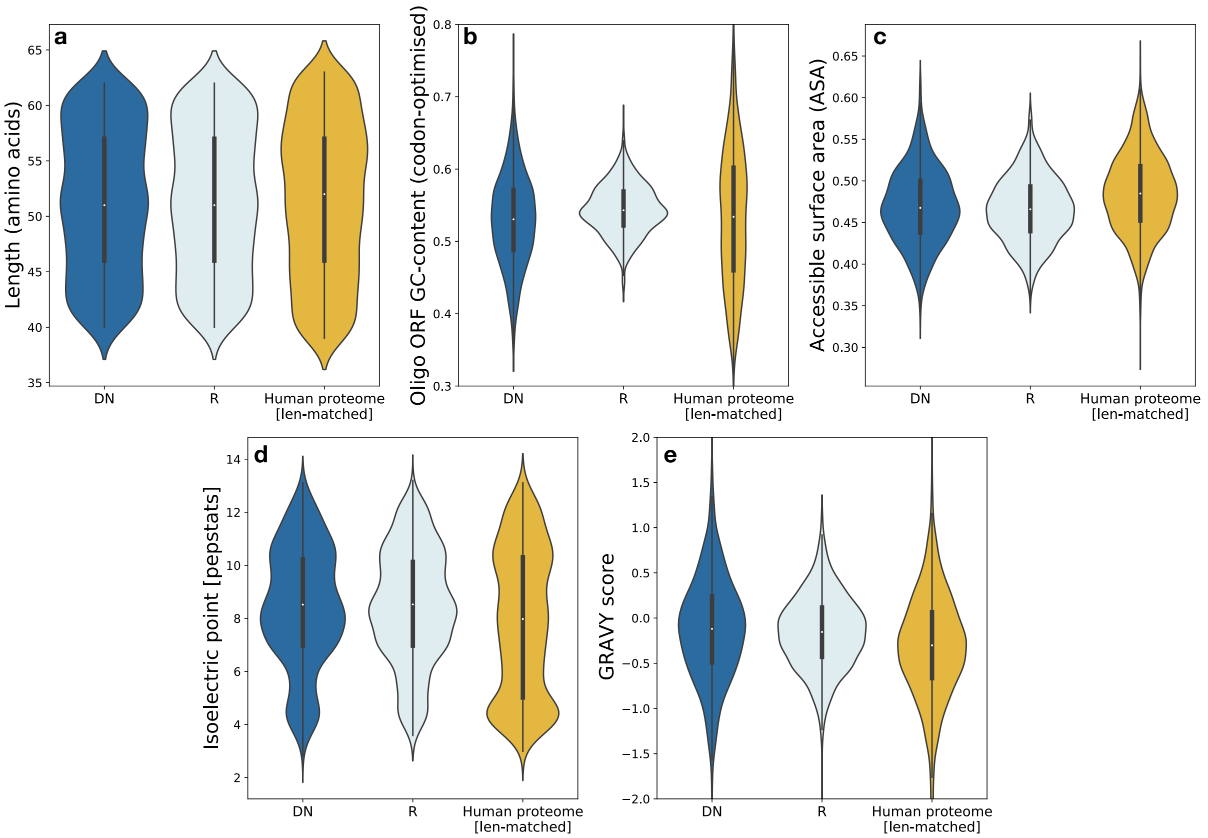

Figure S1: **Predicted sequence properties for a synthesised library of *de novo* and synthetic random sequences.** Distribution of each property shown for library DN (n=1800; dark blue), library R (n=1800; pale blue), and a random, length-distribution matched sample of proteins from the human proteome (n=3600; yellow). Boxes indicate median and interquartile range (IQR), whiskers indicate 1.5 x IQR.

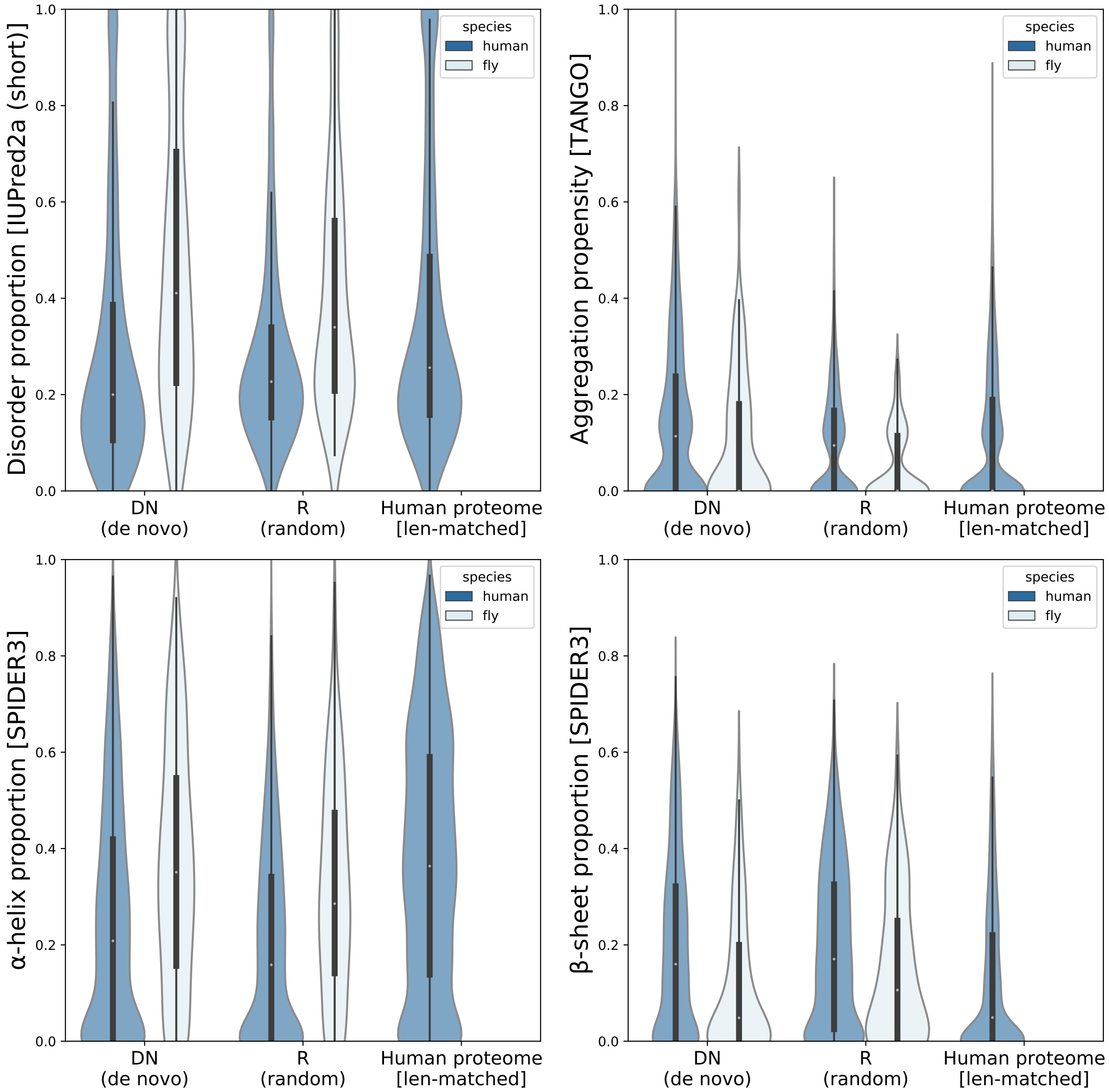

Figure S2: **Predicted sequence properties split by species.** Predicted protein sequence properties are here shown split by species of origin / species composition (for DN and R subsets, respectively). The same set of length-matched human ORFs are shown for comparison. Human/human-like subsets (n=1624) of DN and R are predicted to have lower disorder than fly/fly-like subsets (n=176); length-matched annotated human sequences shown for comparison (n=3600). We attribute this largely to the higher background GC-content of the *Drosophila* genome compared to *H. sapiens*. All pairwise comparisons between human and fly subsets of DN and R were significantly different with small to medium effect sizes (see Table S3). Boxes indicate median and IQR, whiskers indicate 1.5 x IQR.

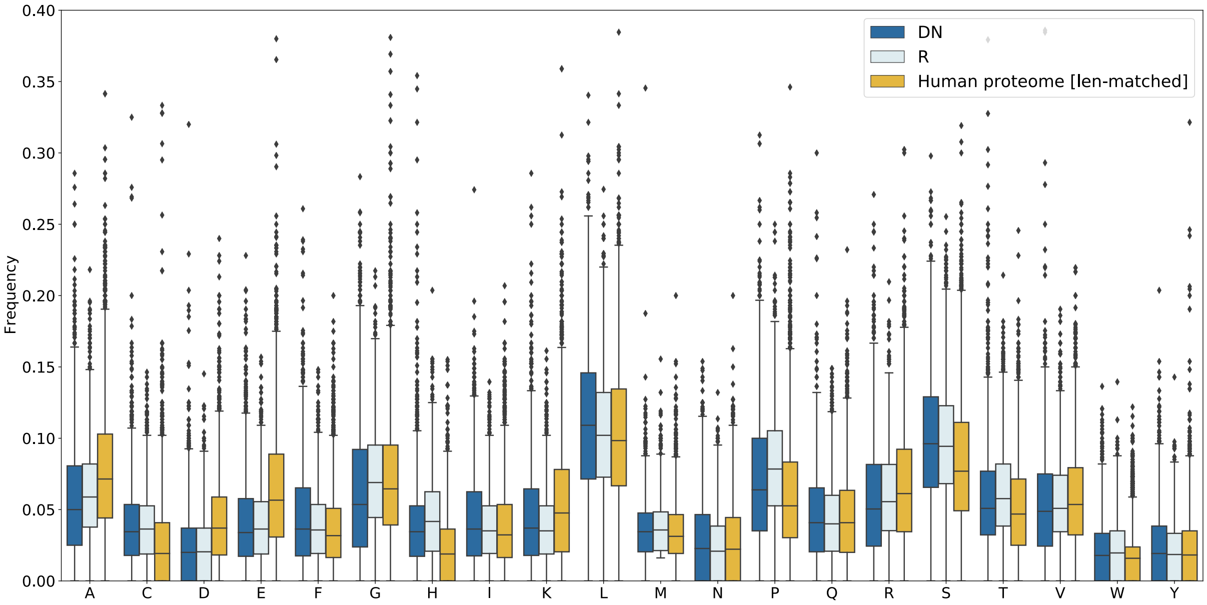

Figure S3: **Amino acid composition of libraries DN and R compared to a set of conserved human proteins.** A synthetic random protein (R, n=1800) library was designed to have closely matched length distribution and amino acid composition as the library of putative *de novo* proteins (DN, n=1800). A length-matched random sample of the annotated human proteome (distinct from the putative human *de novo* proteins in library DN) is shown as a reference (n=3600). Boxes indicate median and IQR, whiskers indicate 1.5 x IQR.

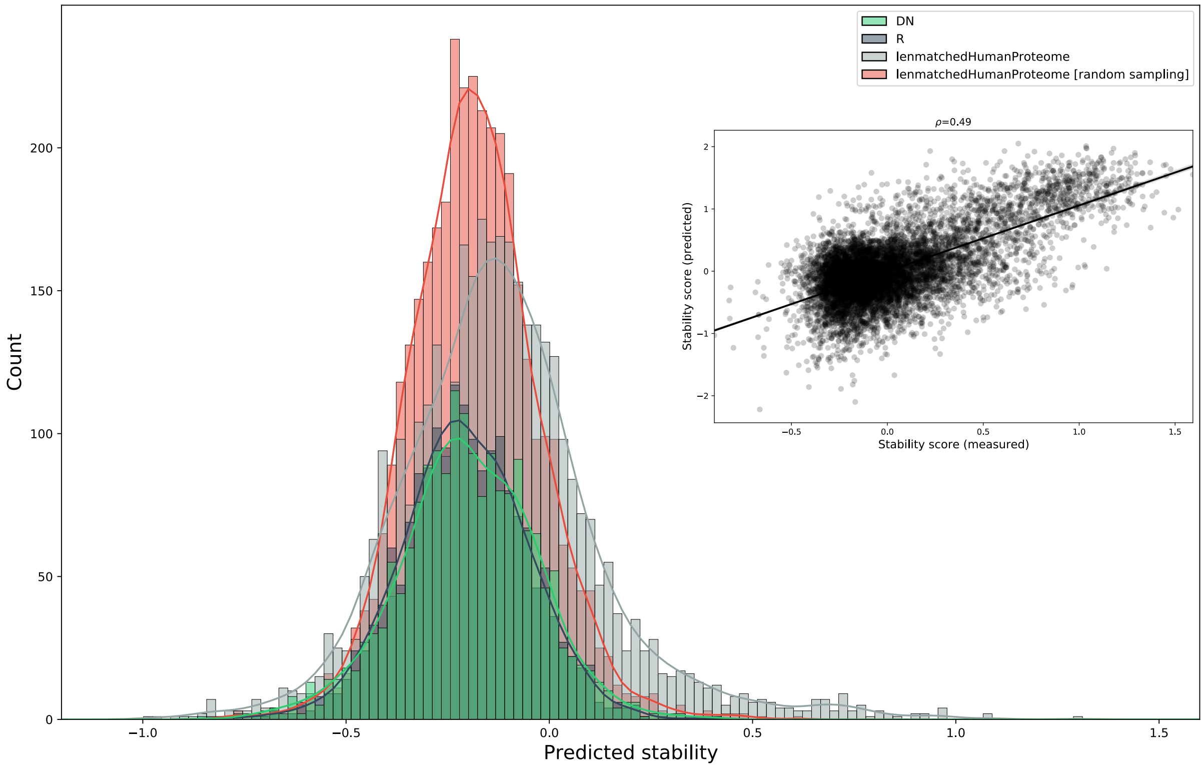

Figure S4: **Prediction of library stability using a learned protein embedding.** The UniRep embedding was used to train a model based on proteolysis-derived stability scores. Using this model, library R (dark grey, n=1800), with length and amino acid frequency distributions identical to library DN (green, n=1800), is predicted to have a similar stability distribution. Predictions for a length-matched subset of the annotated human proteome (grey, n=3600) and for a randomised equivalent (red, n=3600) are shown for comparison. **Inset:** Correlation of predicted and measured stability scores on holdout set after training on experimental dataset of ca. 50,000 *de novo*-designed proteins assayed by Rocklin *et al.* (2020).

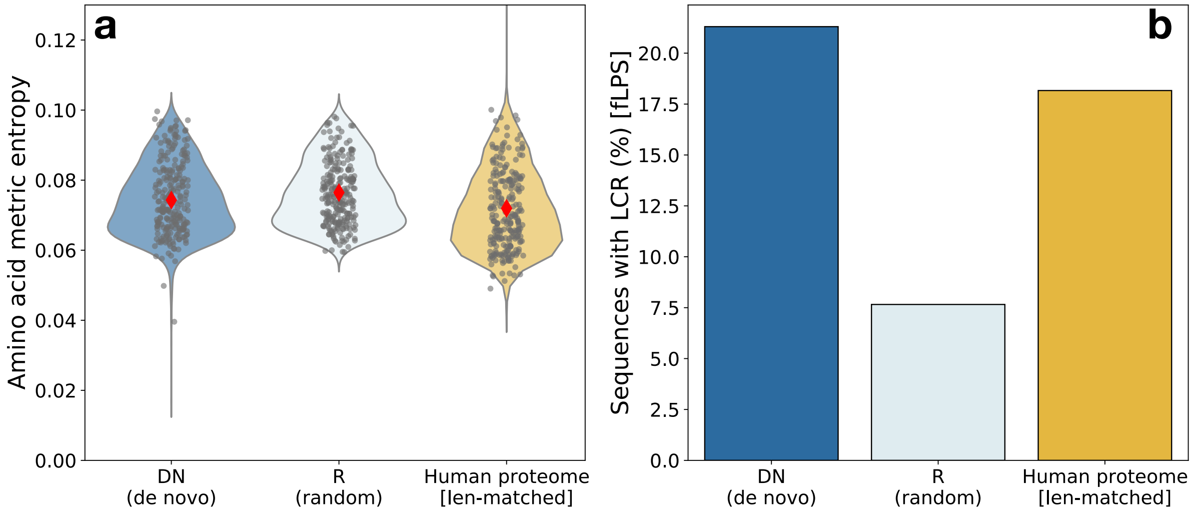

Figure S5: **Amino acid sequence information content.** **a)** To assess sequence information content and the impact of the random sampling approach used to generate the random library subpool (R), per-sequence metric entropy (length-normalised Shannon entropy) was calculated. Overall sequence complexity of library ORFs (DN and R; n=1800) is similar to conserved human ORFs n=3600. Diamonds indicate mean values. **b)** Low complexity regions (LCRs) were also identified for the same sets of sequences, including short compositionally-biased regions that may not have a major effect on the overall sequence complexity. We find that compared to biologically-occurring ORFs (DN and human proteome pools), our random library subpool (R) has a significantly lower number of ORFs harbouring at least one LCR (p=1.39e-31, two-proportions z-test).

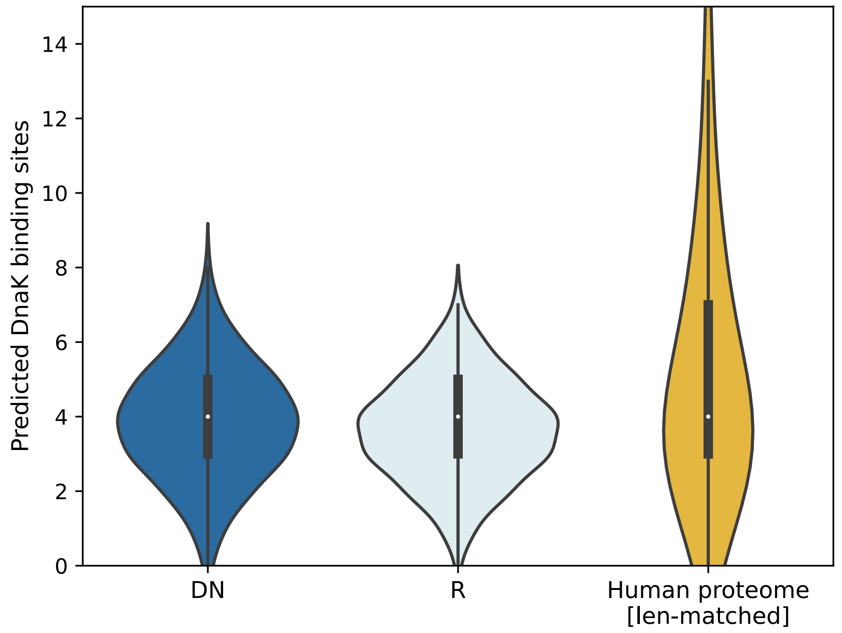

Figure S6: **DnaK binding site prediction.** Bioinformatic prediction of the number of DnaK binding sites per sequence for libraries DN (n=1800) and R (n=1800), with a length-matched set of annotated human proteins included for comparison (n=3600); sequences from libraries DN and R are predicted to have similar DnaK affinity, with median number of predicted sites similar to that of conserved human proteins (ca. four sites per sequence). Boxes indicate median and IQR, whiskers indicate 1.5 x IQR.

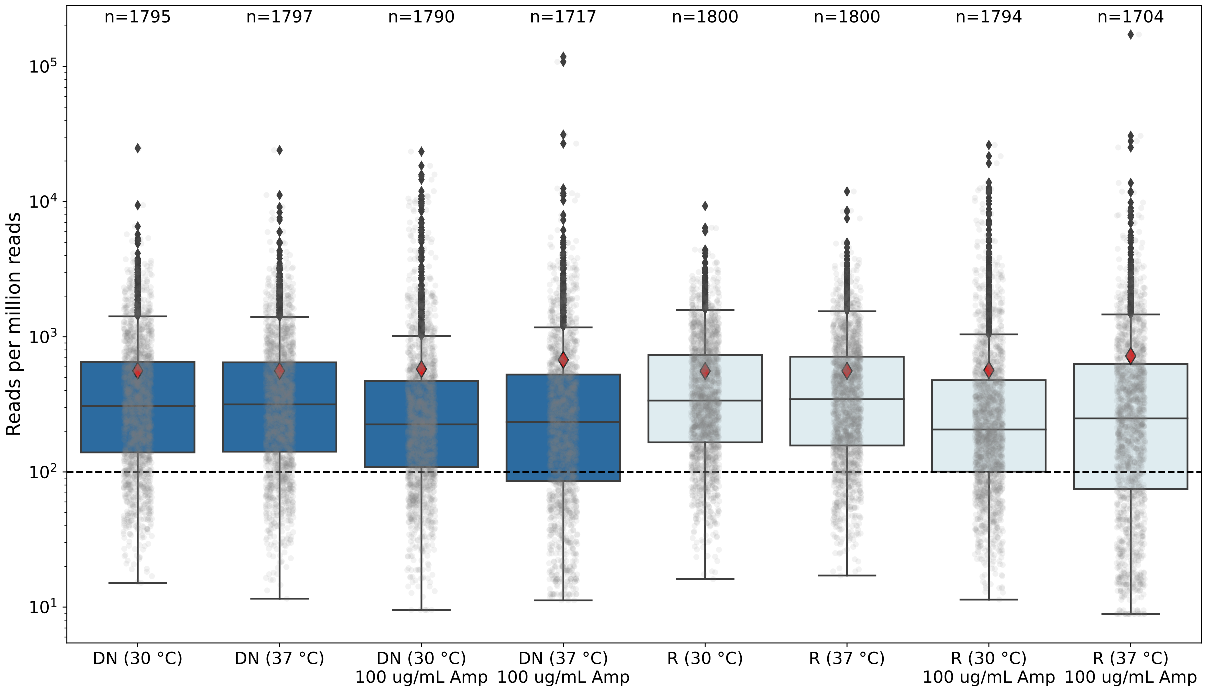

Figure S7: **Twin-arginine export quality assay: NGS read count distributions.** Read counts normalised for sequencing depth across demultiplexed sample subsets. Based on read count distributions, a threshold of 100 reads-per-million (dashed line) was used to remove those variants present at only low levels in the input library. Proportions of each library represented above this threshold are shown in Fig. 3 and Fig. S8. The distribution of read counts was highly similar for libraries DN and R following initial PCR amplification and sub-cloning (i.e. comparing input libraries, without ampicillin), and represented the designed libraries with >99% of variants present after drop-out (DN, 30°C: 1795; DN, 37°C: 1797; R, 30°C: 1800; R, 37°C: 1800). For each condition, the plating assay was repeated with nine replicates. Mean read count per variant across all replicates are shown here; boxes indicate median and IQR of distribution, whiskers indicate 1.5 x IQR. Number of unique variants represented in each sample distribution is annotated above plot.

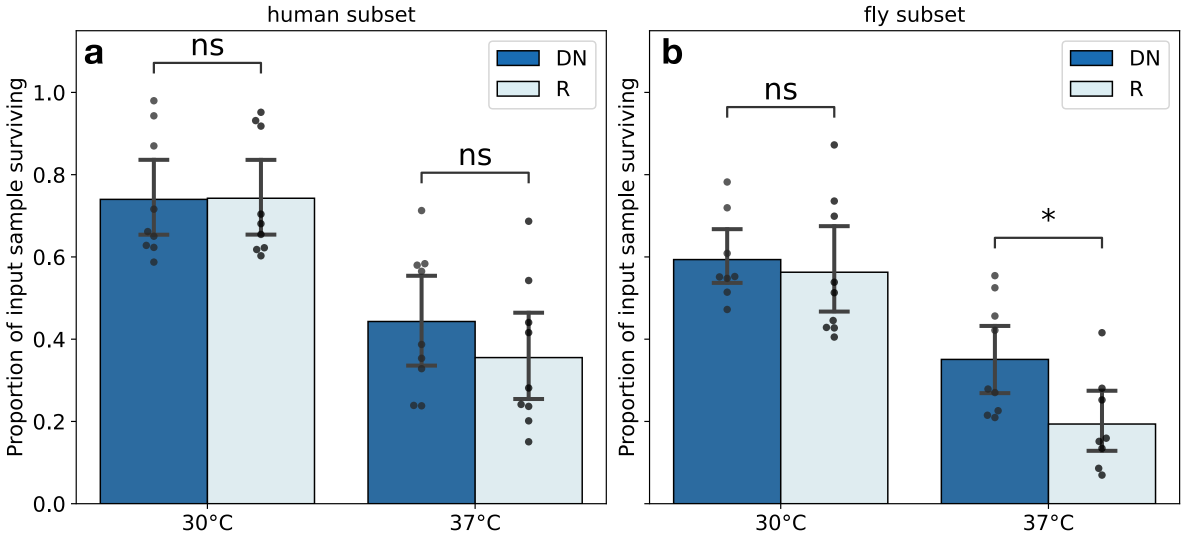

Figure S8: **Twin-arginine assay NGS survival quantification.** **a)** Survival quantification for human library subsets (n=1624) i.e. human putative *de novo* genes (DN) & composition-matched random sequences (R). **b)** Survival quantification for fly library subsets (n=176). **Both panels**: Error bars show 95% confidence intervals around the mean; n=9 (DN, 30 °C), n=9 (R, 30 °C), n=9 (DN, 37 °C), n=8 (R, 37 °C) NGS samples following outlier exclusion. Significance testing is described in Table S4. *; p-value <0.05, ns; not significant; one-sided T-test, unadjusted.

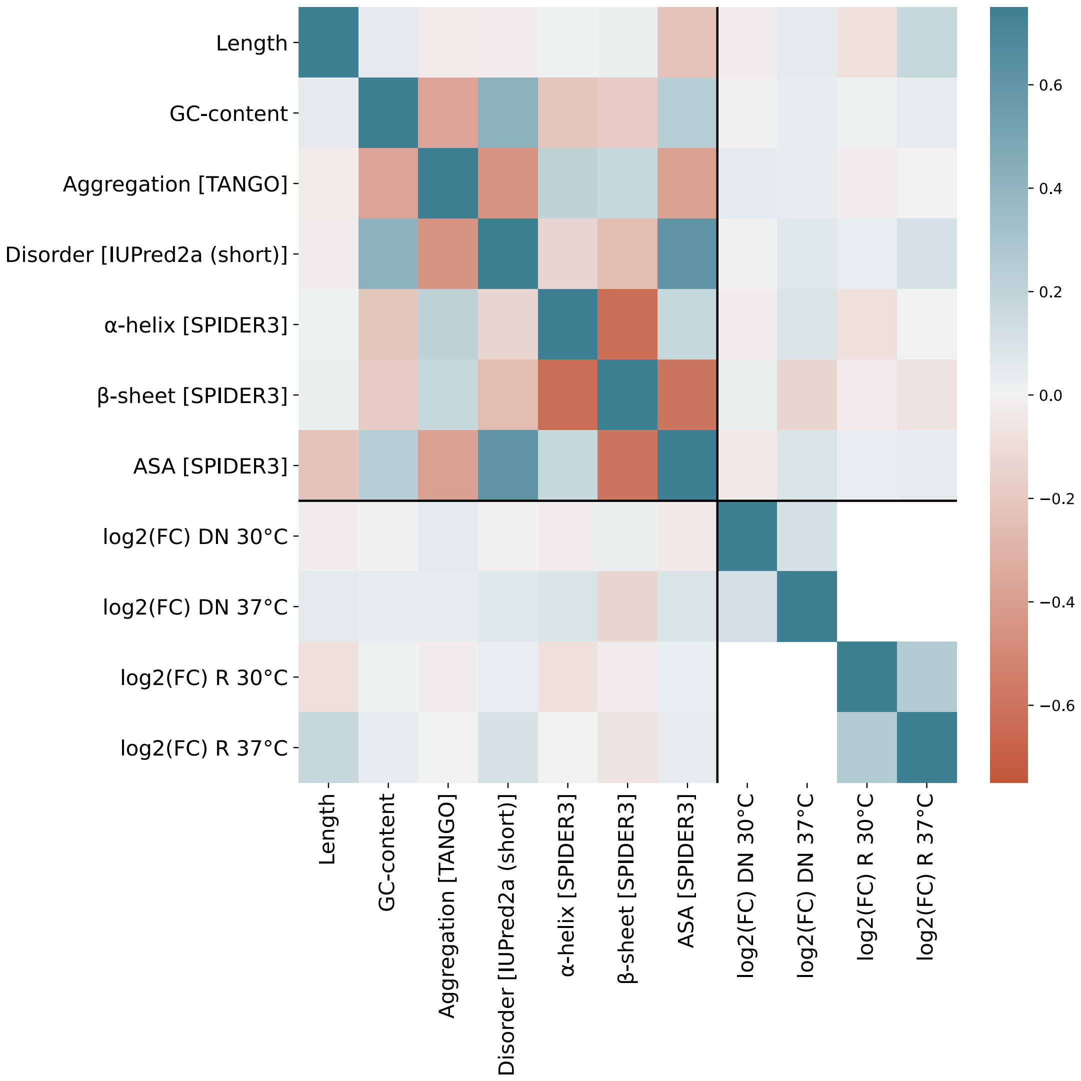

Figure S9: **Correlation matrix of predicted sequence features.** Pearson correlation coefficient between pairs of predicted protein properties indicated by colour. Features are also shown correlated with Twin-arginine assay log2 fold change (i.e. enrichment following selection on ampicillin). No significant correlation was seen between enrichment factors and any sequence feature.

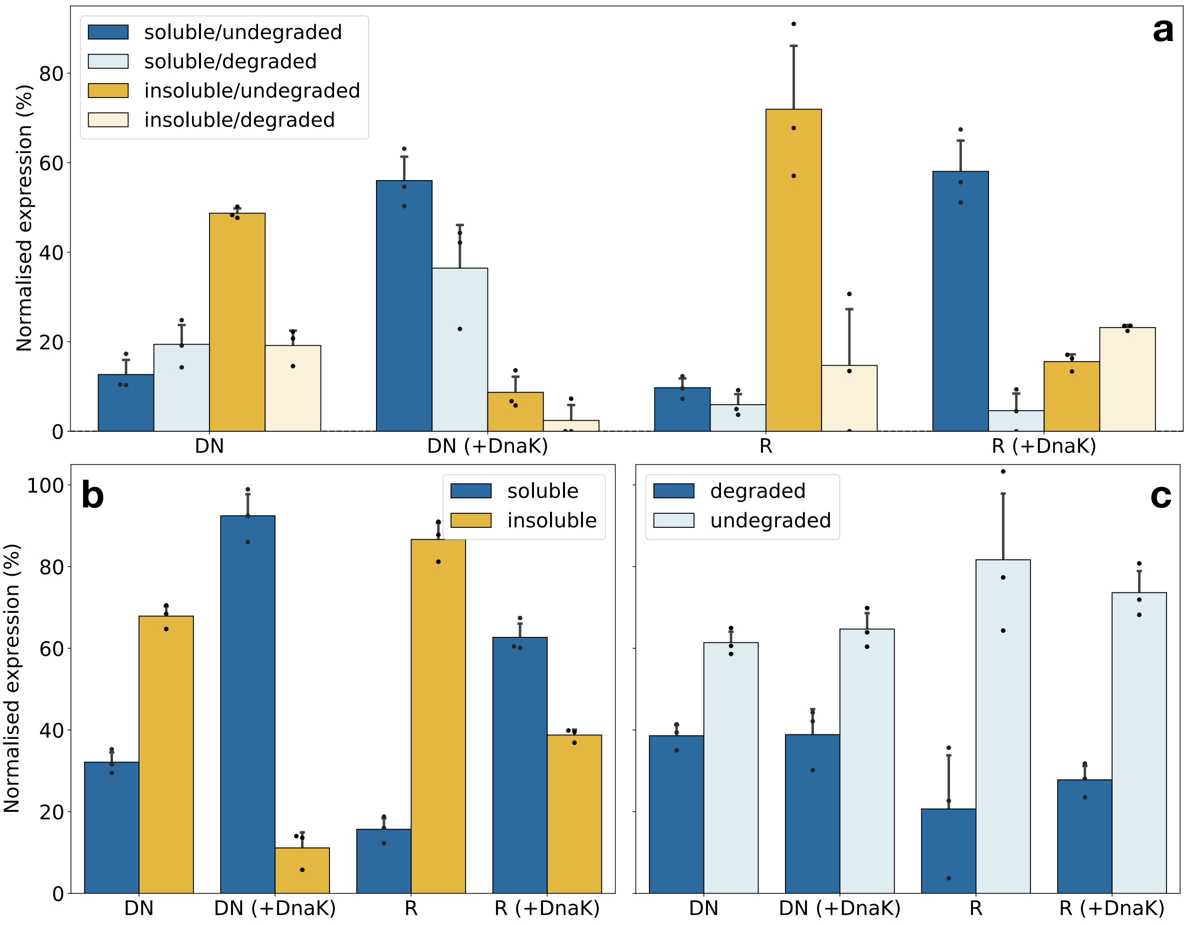

Figure S10: **Quantification of expression from triplicate Western blots with effect of DnaK chaperone and Lon protease addition. a)** Percentage of library protein expression in a cell-free format was estimated by band intensity following anti-FLAG® Western blot. Stacked bars in Fig. 5c/d show mean percentage of expression for each condition across the three replicates. **b)** The same data with soluble and insoluble fractions collapsed. One-tailed T-test indicated DN to be significantly more soluble than R both without (p-value=0.00148) and with (p-value=0.000630) DnaK. **c)** Degraded and undegraded fractions collapsed. One-tailed T-test indicated R to be overall significantly more undegradable than DN (p-value=0.0167) when chaperone and no-chaperone samples are grouped together (individual comparisons non-significant). **All panels**: Error bars indicate standard deviation of three replicates around the mean (n=3).

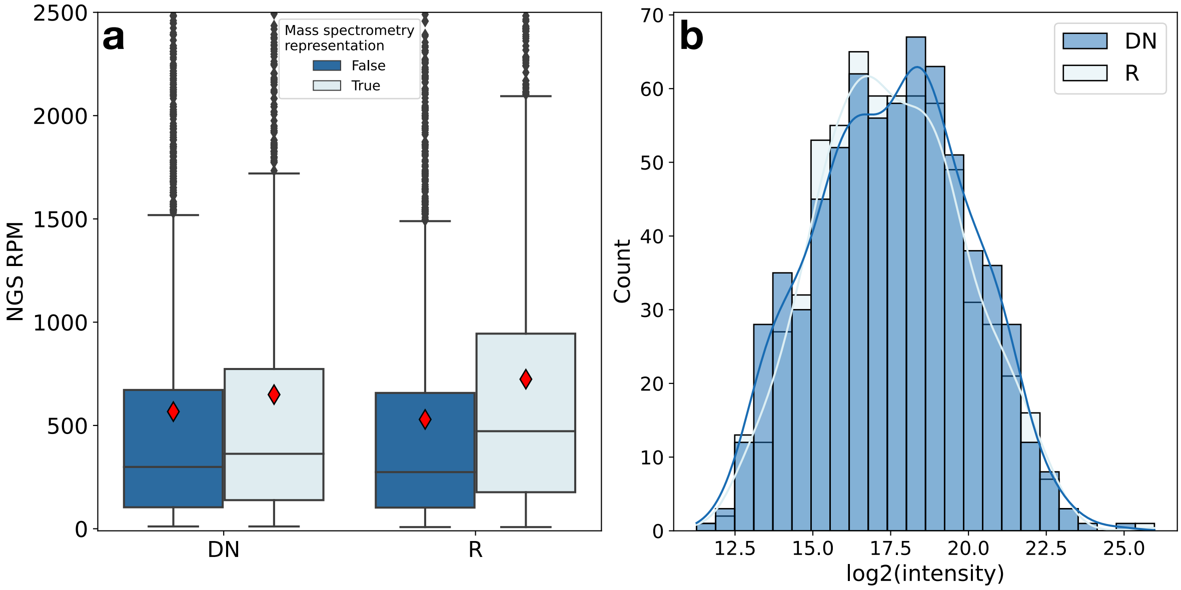

Figure S11: **Quantification of cell-free protein expression by mass spectrometry compared to next-generation sequencing. a)** Distribution of NGS read counts for library subsets without/with cell-free protein expression confirmed by mass spectrometry (MS). For both libraries DN and R, subsets with confirmed protein expression (ca. 1/3 of each library) had higher NGS read counts on average than the remaining synthesised sequences; diamonds indicate mean RPM value (Mann-Whitney U=4.39E-06 (DN), 1.69E-33 (R)). See also Fig. 4b for a breakdown of library subsets with expression identified by NGS and/or MS. **b)** Histogram of mass spectrometry intensities for those proteins detected in each of libraries DN and R. Distributions equal (Mann-Whitney U=6.95E-01). Boxes indicate median and IQR, whiskers indicate 1.5 x IQR.

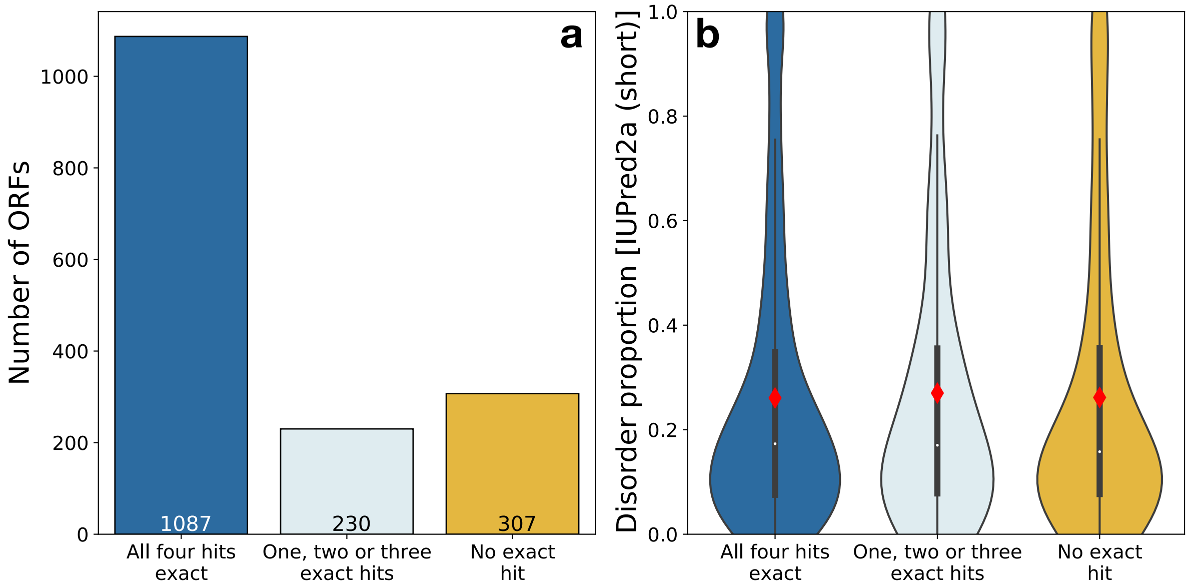

Figure S12: **Human *de novo* ORF conservation.** **a)** To assess conservation of the human subset of library DN (1624/1800 ORFs), we mapped ORFs to consensus genomes of a representative individual from four human populations found in the 1000 Genomes dataset (taking the highest scoring BLAT hit in each genome as its mapping). The four populations used were: CHS (genome HG00650), CLM (genome HG01250), FIN (genome HG00346), GBR (genome HG00251). As a coarse measure of genomic fixation at the population-level, the amino acid sequence identity of each match was calculated, and the 1624 human ORFs were categorised by how many of the four possible hits was an exact amino acid match. ORFs are labelled 'all four hits exact' (perfect match in all four genomes), 'one, two or three exact hits', and 'no exact hit' (no exact matches in the four genomes). While the 1800 short (<66 amino acids) ORFs lack sufficient polymorphic sites in this population data for robust analysis of site-frequency spectra, the fact that over two thirds (1087/1624, 67%) of the *de novo* ORFs appear highly conserved across multiple human populations points to significant genomic conservation. In 157/1624 ORFs, the ORF was found to be disrupted in the best hit(s) across the four populations. However, in the absence of multi-population RNA-Seq data corresponding to the expression conditions analysed by Dowling *et al.* (2020), a more involved investigation of conservation is currently not tractable. **b)** Predicted intrinsic disorder for the human *de novo* subset broken down by ORF mapping category (n=1087/230/307 as in panel (a)) does not reveal any significant trends e.g. for poorly conserved ORFs to be drivers of sequence property trends. Diamonds indicate mean value; differences non-significant (see Table S3). Boxes indicate median and IQR, whiskers indicate 1.5 x IQR.

Library	Error type	Error-free reads (%)
DN	Substitution	99.9
DN	InDel	98.6
R	Substitution	100.0
R	InDel	99.8

Table S1: **Library quality following synthesis**. Next-generation sequencing (NGS) was used to assess the diversity and quality of each library. Error rates calculated independently for substitutions and InDels show that the majority of reads are error free following synthesis and sub-cloning of both libraries DN and R.

Primer	Sequence
DN_F	CTATGA gaattc GAGTCCCATGTACATATG
DN_R	TCATAG ggatcc TGACGACCCCTGCTCGAG
R_F	CTATGA gaattc TGGCTCCATACACATATG
R_R	TCATAG ggatcc CAGAGGAACCCGCTCGAG
pSAL_NSG_F_01	CGTACTAG gactgcgcatatggaattc
pSAL_NSG_F_02	AGGCAGAA gactgcgcatatggaattc
pSAL_NSG_F_03	TCCTGAGC gactgcgcatatggaattc
pSAL_NSG_F_04	TAGGCATG gactgcgcatatggaattc
pSAL_NSG_R_01	TATCCTCT gtttctgggtgggatcc
pSAL_NSG_R_02	CTCTCTAT gtttctgggtgggatcc

Table S2: **Primers used in this study.** DN and R subpool primers (top) were used to amplify each subpool from the total oligonucleotide pool and introduce EcoRI and BamHI restriction sites prior to sub-cloning. 'NGS' primers, used for amplicon preparation prior to Next-Generation Sequencing, encode 8-bp barcodes to allow pooling of multiple conditions in a single sample.

Fig.	Property	Group #1	Group #2	Effect size	p-value
2a	IUPred2a disorder	DN	R	None	*5.11E-04*
2a	IUPred2a disorder	DN	Proteome	None	*6.64E-16*
2a	IUPred2a disorder	R	Proteome	*Small*	*1.99E-07*
2b	TANGO aggregation	DN	R	*Small*	*4.46E-14*
2b	TANGO aggregation	DN	Proteome	*Small*	*8.01E-12*
2b	TANGO aggregation	R	Proteome	None	1.07E-01
2c	SPIDER3 α-helix	DN	R	*Small*	*2.65E-08*
2c	SPIDER3 α-helix	DN	Proteome	*Small*	*2.82E-44*
2c	SPIDER3 α-helix	R	Proteome	*Medium*	*3.04E-91*
2d	SPIDER3 β-sheet	DN	R	None	*3.82E-02*
2d	SPIDER3 β-sheet	DN	Proteome	*Small*	*2.50E-39*
2d	SPIDER3 β-sheet	R	Proteome	*Small*	*9.42E-55*
S1	Length (amino acids)	DN	R	None	4.75E-01
S1	Length (amino acids)	DN	Proteome	None	4.73E-01
S1	Length (amino acids)	R	Proteome	None	4.49E-01
S1	Oligo ORF GC-content	DN	R	*Small*	*3.30E-17*
S1	Oligo ORF GC-content	DN	Proteome	None	6.56E-02
S1	Oligo ORF GC-content	R	Proteome	None	*1.18E-05*
S1	Accessible surface area	DN	R	None	*2.87E-02*
S1	Accessible surface area	DN	Proteome	*Small*	*4.57E-31*
S1	Accessible surface area	R	Proteome	*Small*	*2.43E-46*
S1	Isoelectric point	DN	R	None	5.00E-01
S1	Isoelectric point	DN	Proteome	*Small*	*1.68E-11*
S1	Isoelectric point	R	Proteome	*Small*	*1.43E-12*
S1	GRAVY score	DN	R	None	*3.25E-02*
S1	GRAVY score	DN	Proteome	*Small*	*3.35E-26*
S1	GRAVY score	R	Proteome	*Small*	*5.35E-24*
S2	IUPred2a disorder	DN (human)	DN (fly)	*Medium*	*3.54E-19*
S2	IUPred2a disorder	R (human)	R (fly)	*Medium*	*1.34E-13*
S2	TANGO aggregation	DN (human)	DN (fly)	*Small*	*1.57E-03*
S2	TANGO aggregation	R (human)	R (fly)	*Small*	*6.71E-08*
S2	SPIDER3 α-helix	DN (human)	DN (fly)	*Small*	*2.62E-08*
S2	SPIDER3 α-helix	R (human)	R (fly)	*Medium*	*3.31E-11*
S2	SPIDER3 β-sheet	DN (human)	DN (fly)	*Small*	*3.06E-09*
S2	SPIDER3 β-sheet	R (human)	R (fly)	*Small*	*6.99E-04*
S5a	Amino acid metric entropy	DN	R	*Small*	*1.21E-10*
S5a	Amino acid metric entropy	DN	Proteome	None	*5.85E-20*
S5a	Amino acid metric entropy	R	Proteome	*Small*	*7.45E-56*
S6	DnaK binding sites	DN	R	None	*8.41E-6*
S6	DnaK binding sites	DN	Proteome	*Medium*	*1.91E-14*
S6	DnaK binding sites	R	Proteome	*Medium*	*8.10E-24*
S11a	NGS RPM	DN (with MS)	DN (no MS)	None	*4.39E-06*
S11a	NGS RPM	R (with MS)	R (no MS)	*Small*	*1.69E-31*
S11b	log2(intensity)	DN	R	None	6.95E-01
S12b	IUPred2a (short) disorder	4 exact	1/2/3 exact	None	4.29E-01
S12b	IUPred2a (short) disorder	4 exact	0 exact	None	2.68E-01
S12b	IUPred2a (short) disorder	1/2/3 exact	0 exact	None	2.82E-01

Table S3: **Effect size and significance for property distribution comparisons.** Effect size was assessed by mapping Cohen's D values to thresholds of 0.2 (small effect), 0.5 (medium effect) and 0.8 (large effect). p-values calculated by Mann-Whitney U test. Effect sizes and p-values shown in italics indicate significance.

Group #1	Group #2	p-value
DN (37 °C)	R (37 °C)	*4.92E-02*
DN (30 °C)	R (30 °C)	4.20E-01
DN (human subset, 37 °C)	R (human subset, 37 °C)	1.49E-01
DN (human subset, 30 °C)	R (human subset, 30 °C)	4.53E-01
DN (fly subset, 37 °C)	R (fly subset, 37 °C)	*1.21E-02*
DN (fly subset, 30 °C)	R (fly subset, 30 °C)	2.25E-01

Table S4: **Significance testing for Twin-arginine assay survival.** Significance testing for library DN to have higher survival upon ampicillin selection, as assessed by NGS. Number of unique variants with >100 RMP were compared without and with ampicillin for each replicate (n>=8), with survival defined as proportion of unique variants present without ampicillin that were also detected in the presence of ampicillin (one-tailed T-test p-values following exclusion of outliers more than two standard deviations removed from the mean; italicised values <0.05).
