# Supplementary material for: Experimental characterization of de novo proteins and their unevolved random-sequence counterparts
Source: Nat Ecol Evol. 2023 Apr 6;7(4):570–80. doi: 10.1038/s41559-023-02010-2 (PMC10089919; doi:10.1038/s41559-023-02010-2)
Supplement: Supplementary file 2 — Reporting Summary [file 41559_2023_2010_MOESM2_ESM.pdf]

nature portfolio

Corresponding author(s): Klara Hlouchova

Last updated by author(s): Jan 24, 2023

Reporting Summary

Nature Portfolio wishes to improve the reproducibility of the work that we publish. This form provides structure for consistency and transparency in reporting. For further information on Nature Portfolio policies, see our Editorial Policies and the Editorial Policy Checklist.

Statistics

For all statistical analyses, confirm that the following items are present in the figure legend, table legend, main text, or Methods section.

n/a	Confirmed	
☐	☒	The exact sample size (*n*) for each experimental group/condition, given as a discrete number and unit of measurement
☐	☒	A statement on whether measurements were taken from distinct samples or whether the same sample was measured repeatedly
☐	☒	The statistical test(s) used AND whether they are one- or two-sided<br>*Only common tests should be described solely by name; describe more complex techniques in the Methods section.*
☐	☒	A description of all covariates tested
☐	☒	A description of any assumptions or corrections, such as tests of normality and adjustment for multiple comparisons
☐	☒	A full description of the statistical parameters including central tendency (e.g. means) or other basic estimates (e.g. regression coefficient) AND variation (e.g. standard deviation) or associated estimates of uncertainty (e.g. confidence intervals)
☐	☒	For null hypothesis testing, the test statistic (e.g. *F*, *t*, *r*) with confidence intervals, effect sizes, degrees of freedom and *P* value noted<br>*Give P values as exact values whenever suitable.*
☒	☐	For Bayesian analysis, information on the choice of priors and Markov chain Monte Carlo settings
☒	☐	For hierarchical and complex designs, identification of the appropriate level for tests and full reporting of outcomes
☐	☒	Estimates of effect sizes (e.g. Cohen's *d*, Pearson's *r*), indicating how they were calculated

Our web collection on statistics for biologists contains articles on many of the points above.

Software and code

Policy information about availability of computer code

Data collection	No software was used as part of data collection for this study.
Data analysis	Custom Python code was used to design the libraries studied in the manuscript and analyse all data described in the paper. Library design code is made available (https://zivgitlab.uni-muenster.de/ag-ebb/de-novo/de_novo_lib) as a repository along with example data. Code used to process NGS data is deposited along with the raw data under Zenodo DOI 10.5281/zenodo.7556935, as well as instructions for use. Sequence predictions were made using open source software as described in the Methods section. Statistical analysis and figures were made using custom Python code. Image quantification was carried out in ImageJ. Mass spectrometry data was processed using Andromeda integrated in a MaxQuant environment v1.6.17.0, and Perseus v2.0.7.

For manuscripts utilizing custom algorithms or software that are central to the research but not yet described in published literature, software must be made available to editors and reviewers. We strongly encourage code deposition in a community repository (e.g. GitHub). See the Nature Portfolio guidelines for submitting code & software for further information.

Data

Policy information about availability of data

All manuscripts must include a data availability statement. This statement should provide the following information, where applicable:

- Accession codes, unique identifiers, or web links for publicly available datasets
- A description of any restrictions on data availability
- For clinical datasets or third party data, please ensure that the statement adheres to our policy

Library sequences, twin-arginine assay sequencing reads and processed data files necessary to reproduce the paper are deposited under Zenodo DOI 10.5281/zenodo.7556935

Human research participants

Policy information about studies involving human research participants and Sex and Gender in Research.

Reporting on sex and gender	*Use the terms sex (biological attribute) and gender (shaped by social and cultural circumstances) carefully in order to avoid confusing both terms. Indicate if findings apply to only one sex or gender; describe whether sex and gender were considered in study design whether sex and/or gender was determined based on self-reporting or assigned and methods used. Provide in the source data disaggregated sex and gender data where this information has been collected, and consent has been obtained for sharing of individual-level data; provide overall numbers in this Reporting Summary. Please state if this information has not been collected. Report sex- and gender-based analyses where performed, justify reasons for lack of sex- and gender-based analysis.*
Population characteristics	*Describe the covariate-relevant population characteristics of the human research participants (e.g. age, genotypic information, past and current diagnosis and treatment categories). If you filled out the behavioural & social sciences study design questions and have nothing to add here, write "See above."*
Recruitment	*Describe how participants were recruited. Outline any potential self-selection bias or other biases that may be present and how these are likely to impact results.*
Ethics oversight	*Identify the organization(s) that approved the study protocol.*

Note that full information on the approval of the study protocol must also be provided in the manuscript.

Field-specific reporting

Please select the one below that is the best fit for your research. If you are not sure, read the appropriate sections before making your selection.

☒ Life sciences ☐ Behavioural & social sciences ☐ Ecological, evolutionary & environmental sciences

For a reference copy of the document with all sections, see nature.com/documents/nr-reporting-summary-flat.pdf

Life sciences study design

All studies must disclose on these points even when the disclosure is negative.

Sample size	Sample sizes were not pre-determined using statistical methods, and as large sample sizes were used as was experimentally feasible given the available time and resources.
Data exclusions	No data was excluded from any analyses.
Replication	Attempts at replication were successful for the key results presented in the manuscript. Up to 10 biological replicates are provided for the Twin-arginine export assay, while Western blots for quantification were carried out in triplicate, providing the necessary statistical significance in both cases.
Randomization	Experimental groups (libraries DN and R) were not subject to randomization in this study: the library design process is described fully in the manuscript. In a number of figures, a comparison group made up of protein sequences drawn from the human proteome is also shown. In this case, the amino acid length distribution of this group was matched to that of both libraries DN and R by random sampling after assigning all human proteome sequences to length bins, thereby controlling for sequence length in the presented distributions of biophysical properties.
Blinding	Blinding was not relevant for this study.

Reporting for specific materials, systems and methods

We require information from authors about some types of materials, experimental systems and methods used in many studies. Here, indicate whether each material, system or method listed is relevant to your study. If you are not sure if a list item applies to your research, read the appropriate section before selecting a response.

Materials & experimental systems

n/a	Involved in the study
☐	☒ Antibodies
☒	☐ Eukaryotic cell lines
☒	☐ Palaeontology and archaeology
☒	☐ Animals and other organisms
☒	☐ Clinical data
☒	☐ Dual use research of concern

Methods

n/a	Involved in the study
☒	☐ ChIP-seq
☒	☐ Flow cytometry
☒	☐ MRI-based neuroimaging

Antibodies

Antibodies used	Sigma-Aldrich Monoclonal ANTI-FLAG® M2-Peroxidase (HRP) antibody, A8592
Validation	References for usage of this antibody for similar applications are given in the corresponding datasheet: https://www.sigmaaldrich.com/deepweb/assets/sigmaaldrich/product/documents/111/306/a8592dat-mk.pdf
