# Supplementary material for: Experimental characterization of de novo proteins and their unevolved random-sequence counterparts
Source: Nat Ecol Evol. 2023 Apr 6;7(4):570–80. doi: 10.1038/s41559-023-02010-2 (PMC10089919; doi:10.1038/s41559-023-02010-2)

Figure 4; library DN; merged JPEG

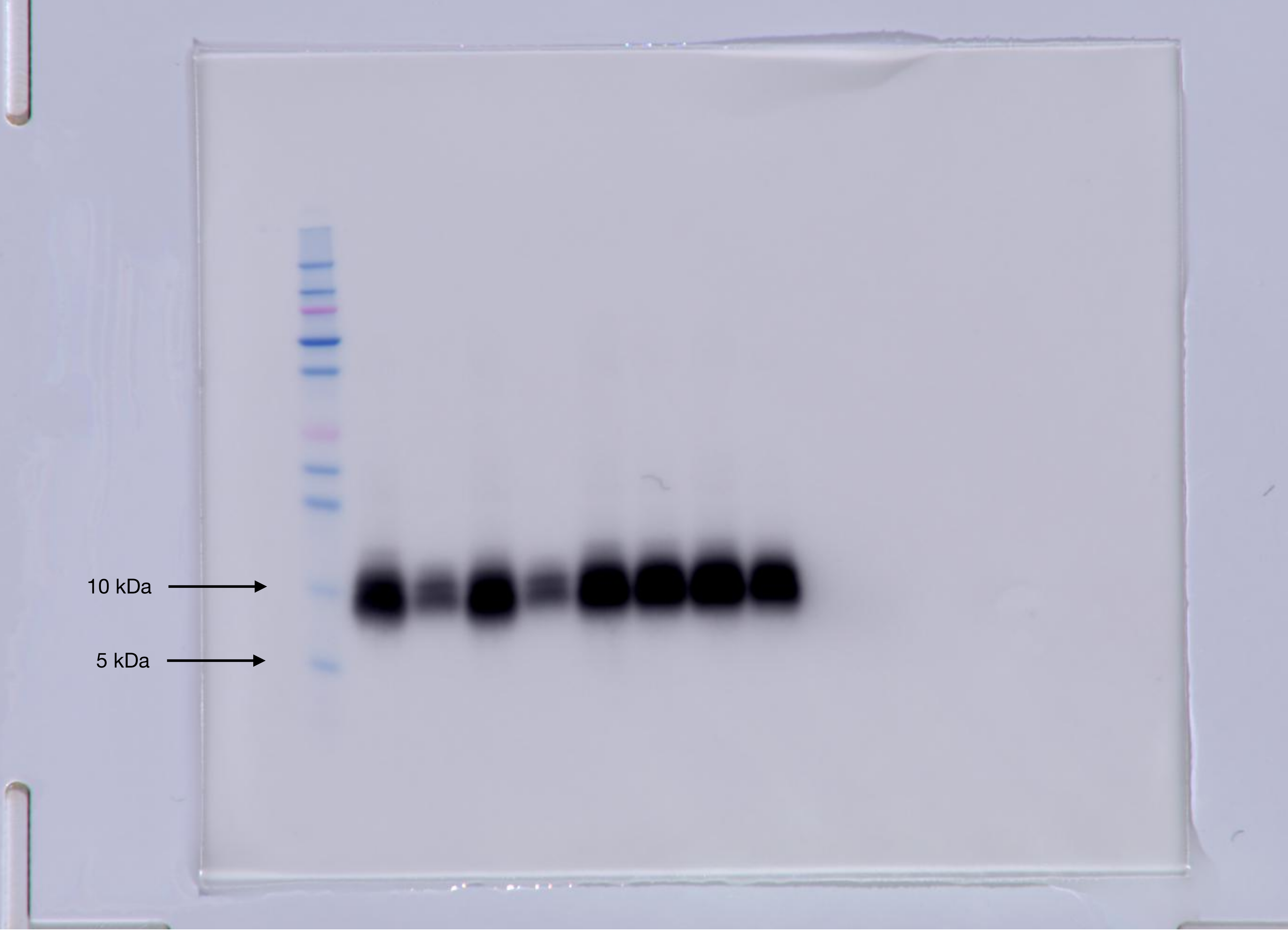

Figure 4; library DN; TIFF

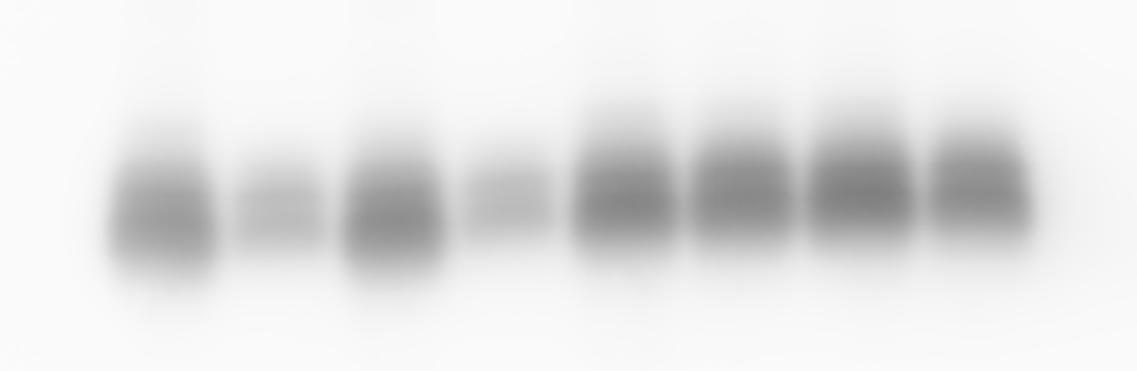

Figure 4; library R; merged JPEG

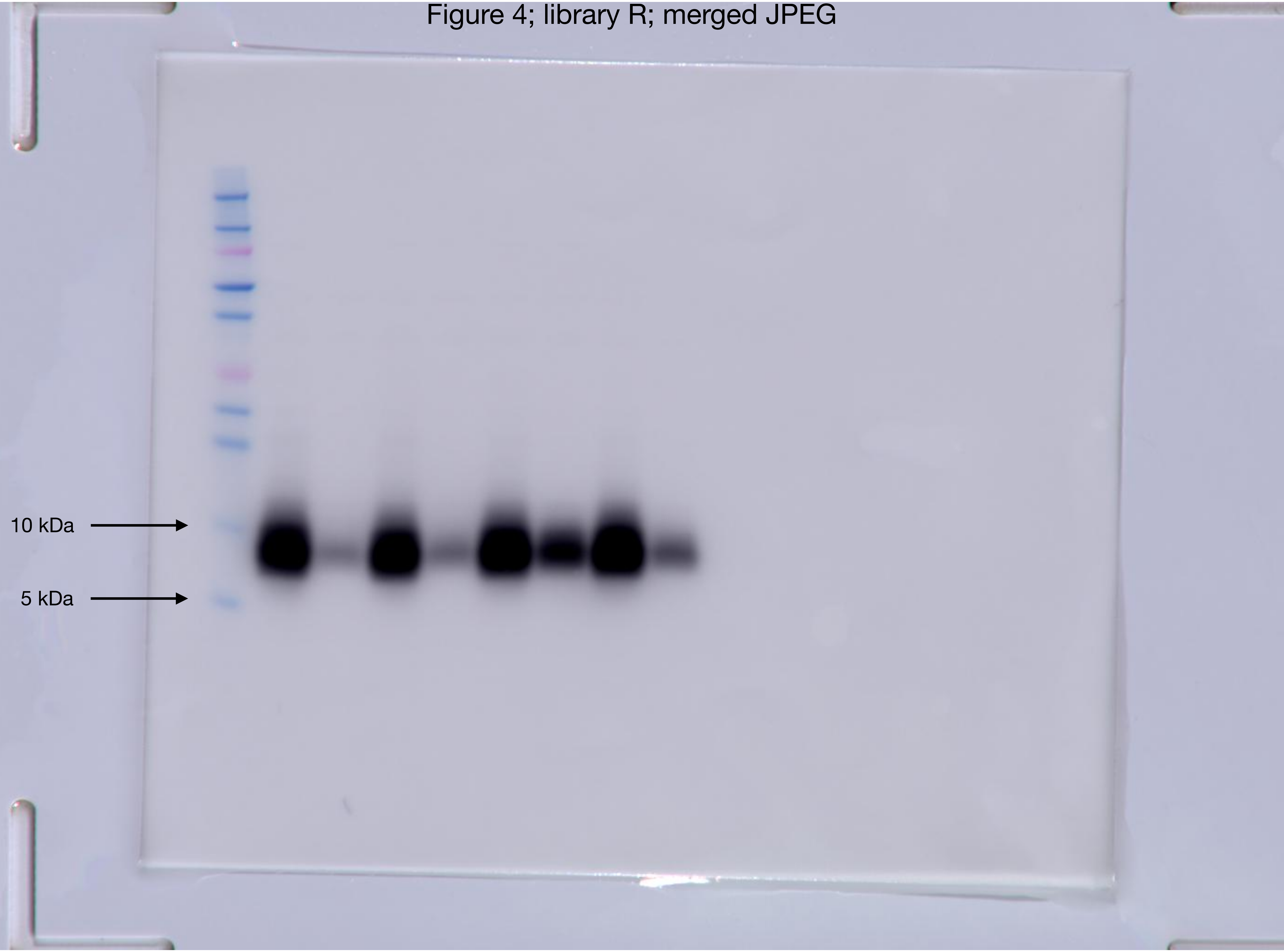

Figure 4; library R; TIFF

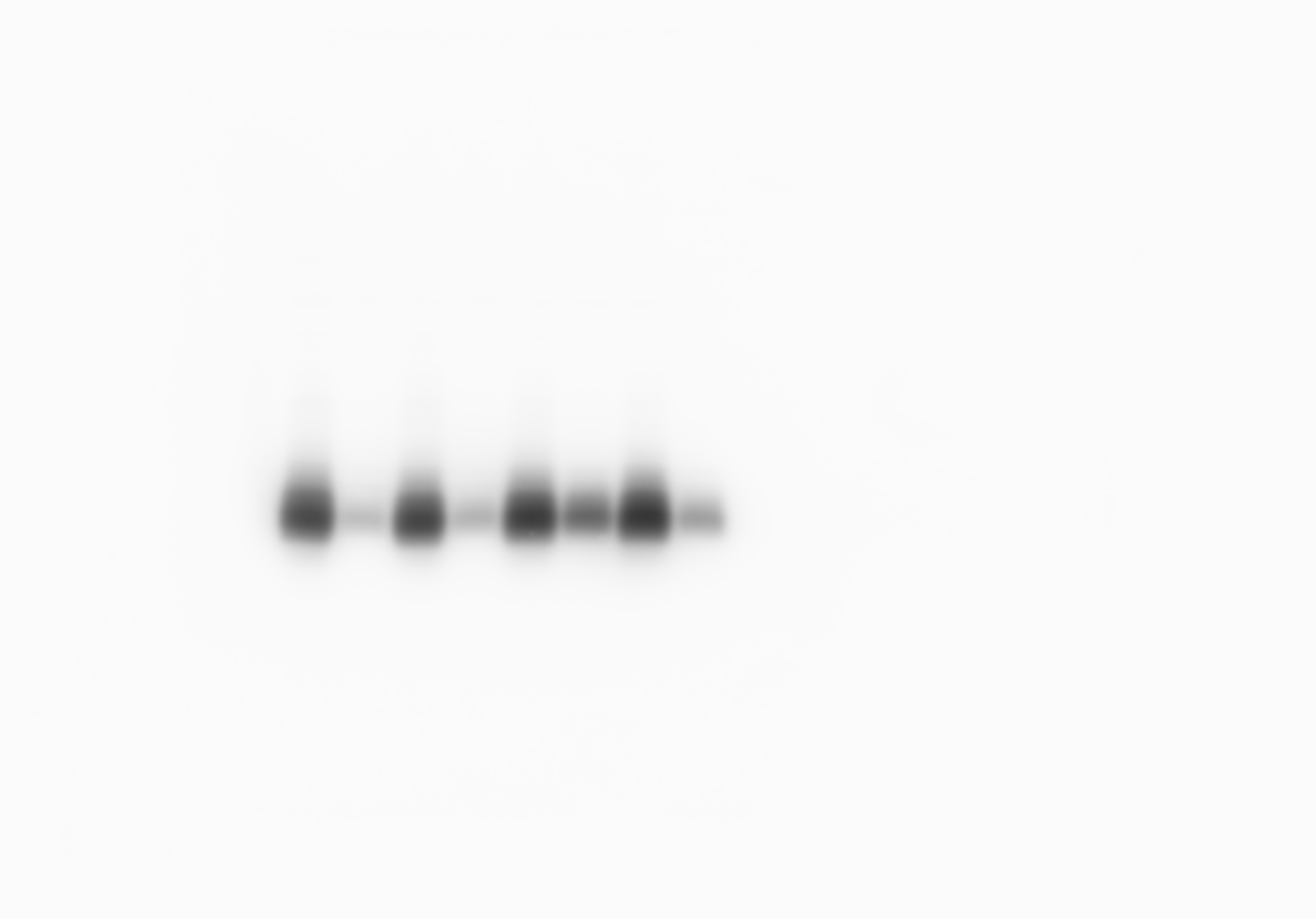

Supplement: Source Data Fig. 4 — Unprocessed western blots. [file 41559_2023_2010_MOESM3_ESM.pdf]
