# Supplementary material for: Experimental characterization of de novo proteins and their unevolved random-sequence counterparts
Source: Nat Ecol Evol. 2023 Apr 6;7(4):570–80. doi: 10.1038/s41559-023-02010-2 (PMC10089919; doi:10.1038/s41559-023-02010-2)

Figure 5; library DN, replicate 1; merged JPEG

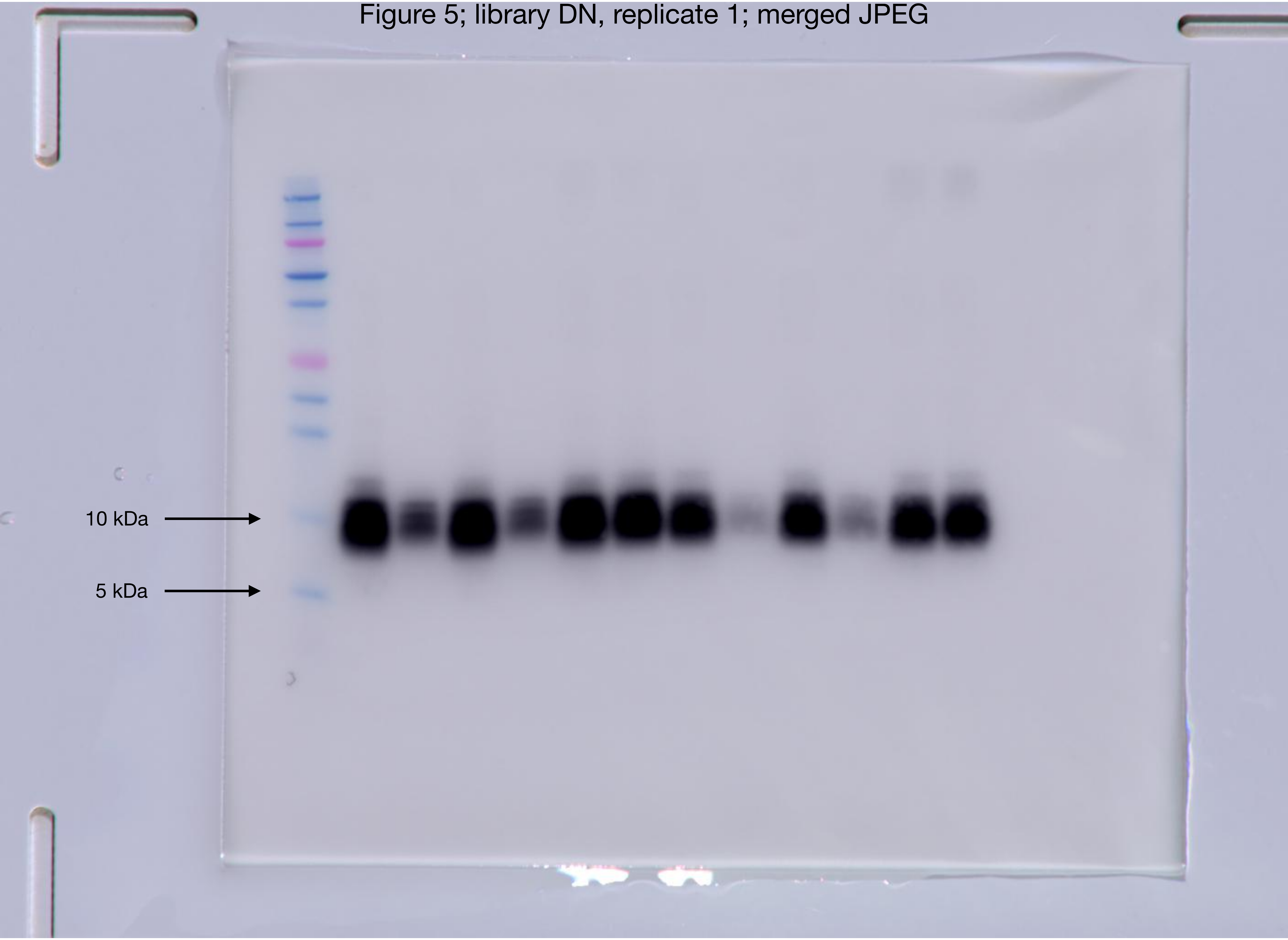

Figure 5; library DN, replicate 1; TIFF

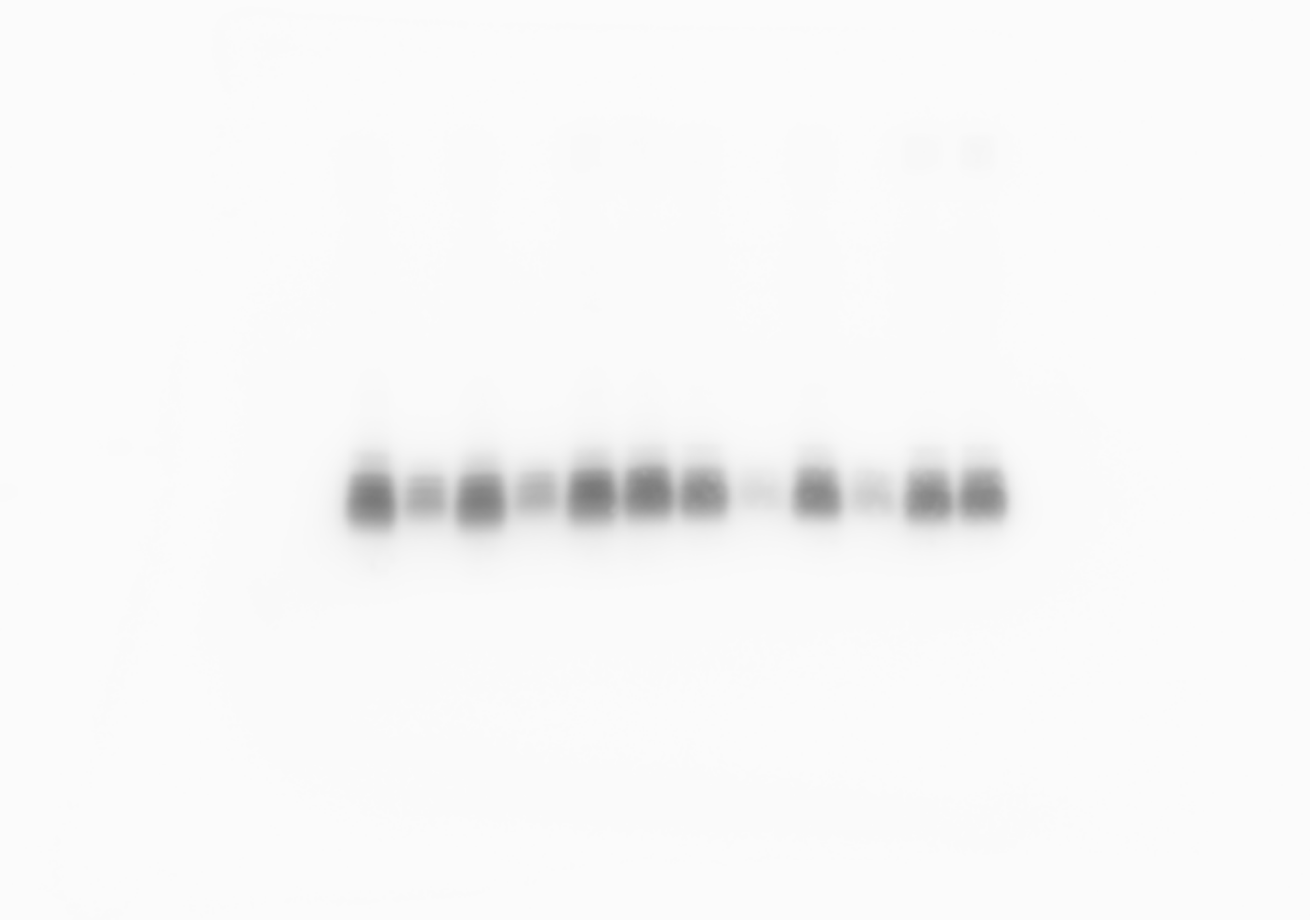

Figure 5; library DN, replicate 2; TIFF

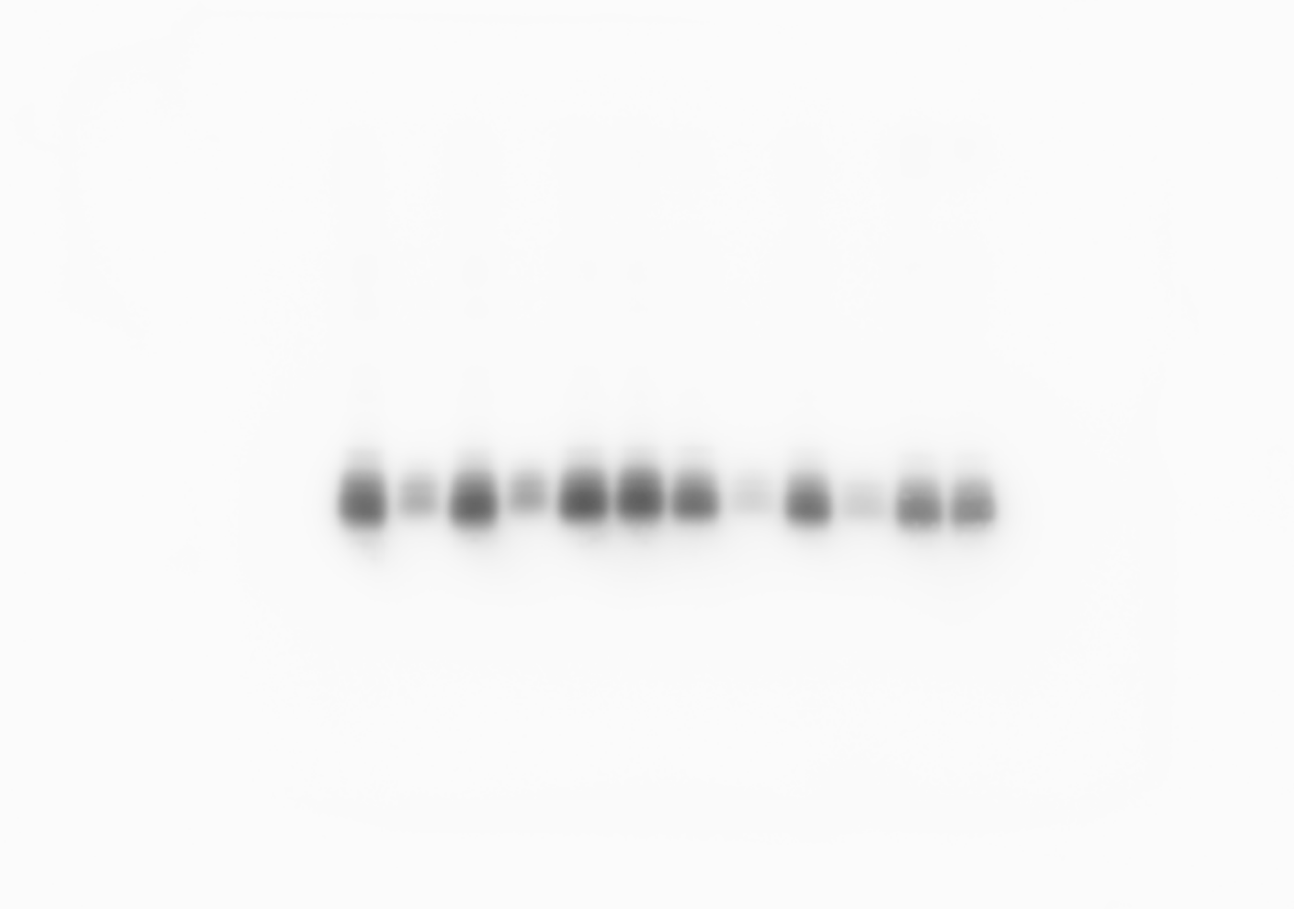

Figure 5; library DN, replicate 3; TIFF

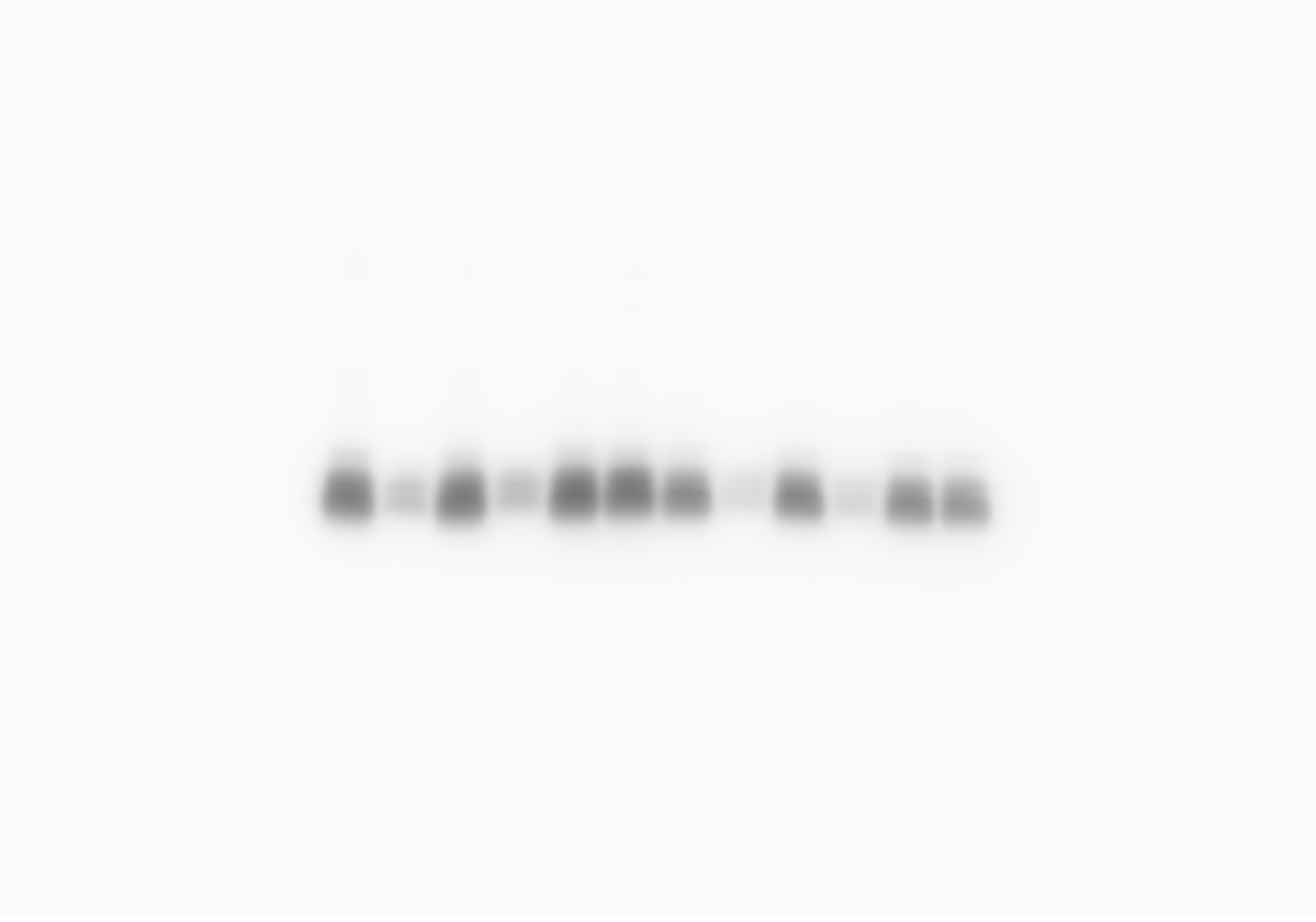

Figure 5; library R, replicate 1; merged JPEG

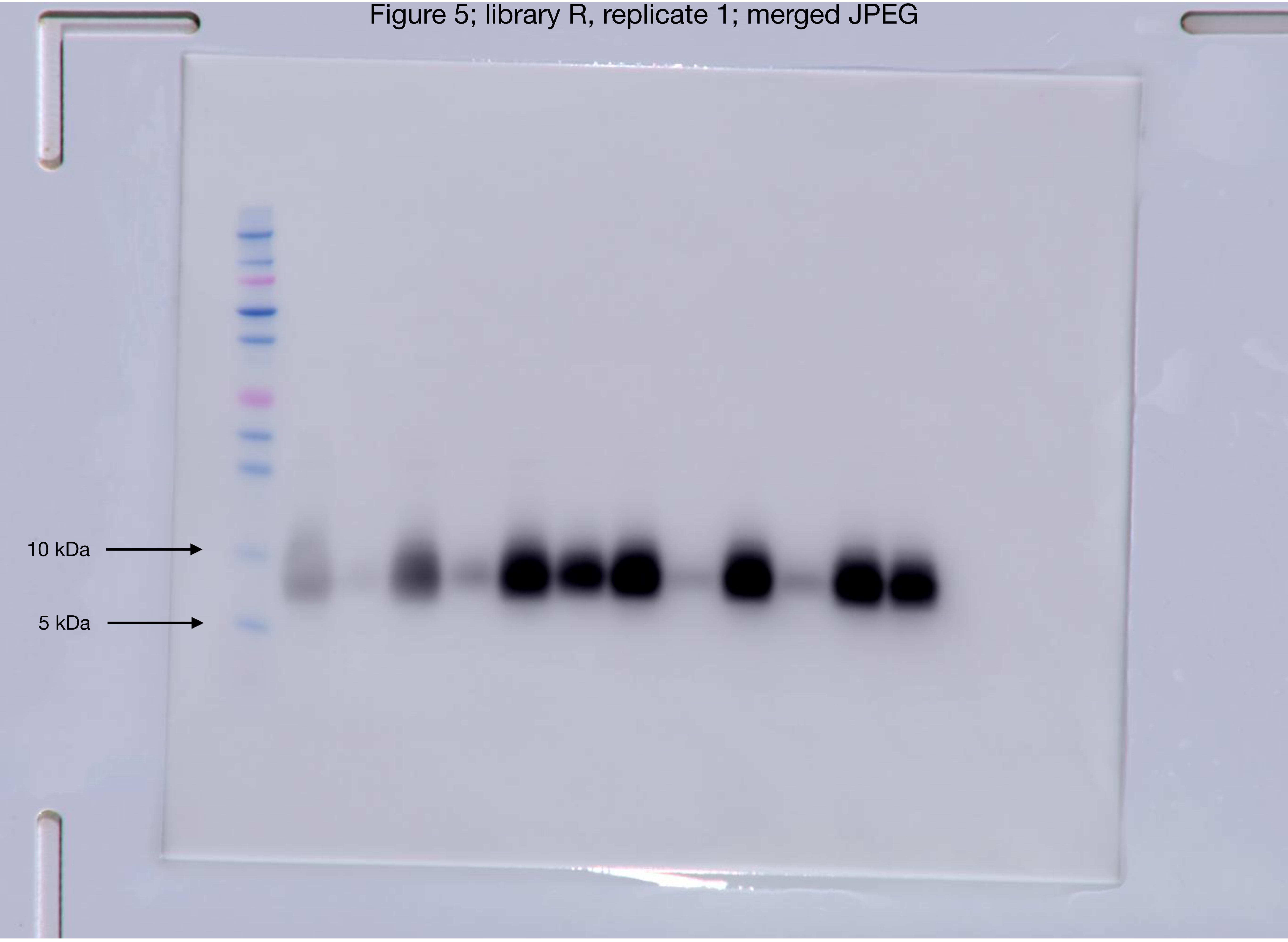

Figure 5; library R, replicate 1; TIFF

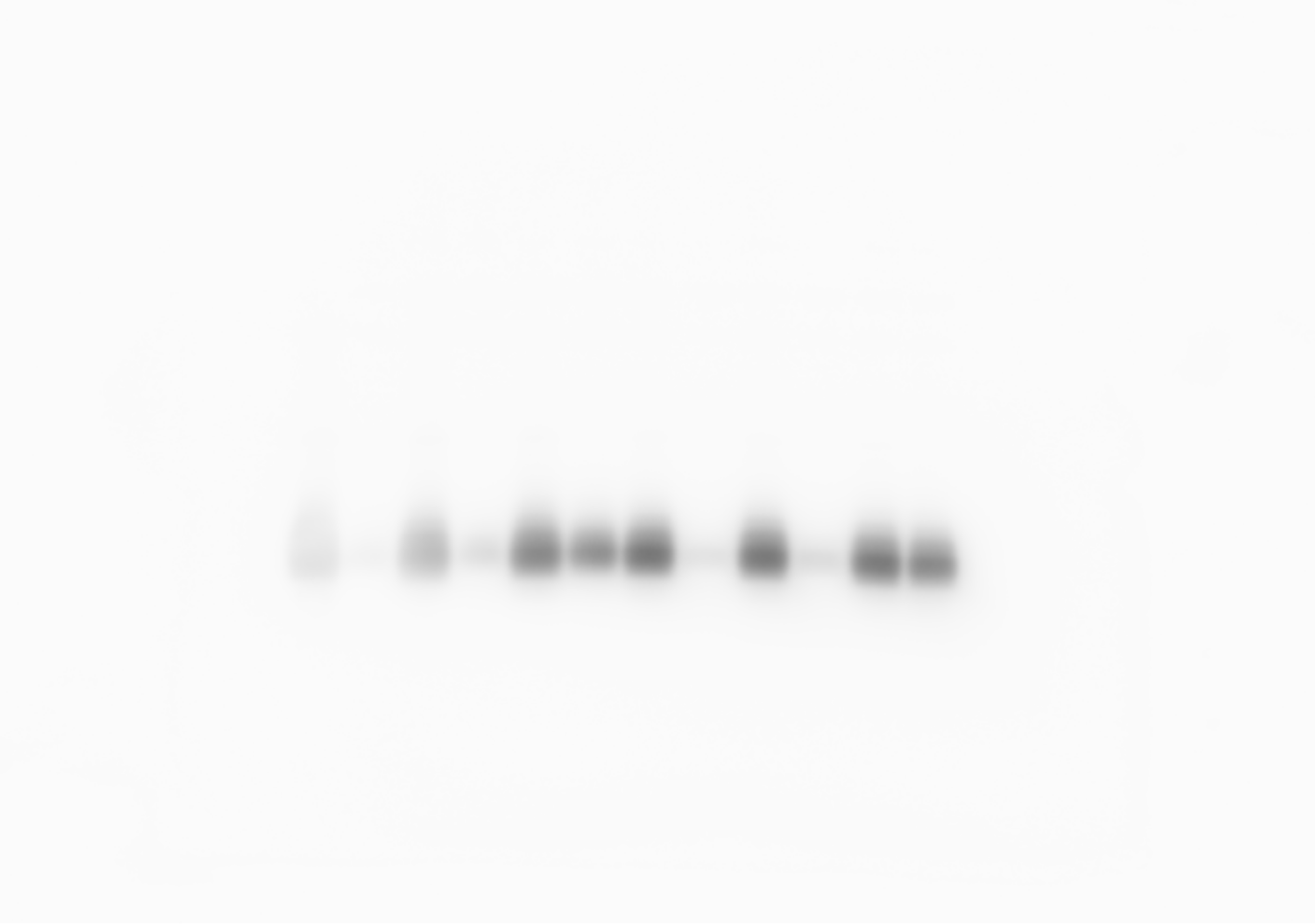

Figure 5; library R, replicate 2; TIFF

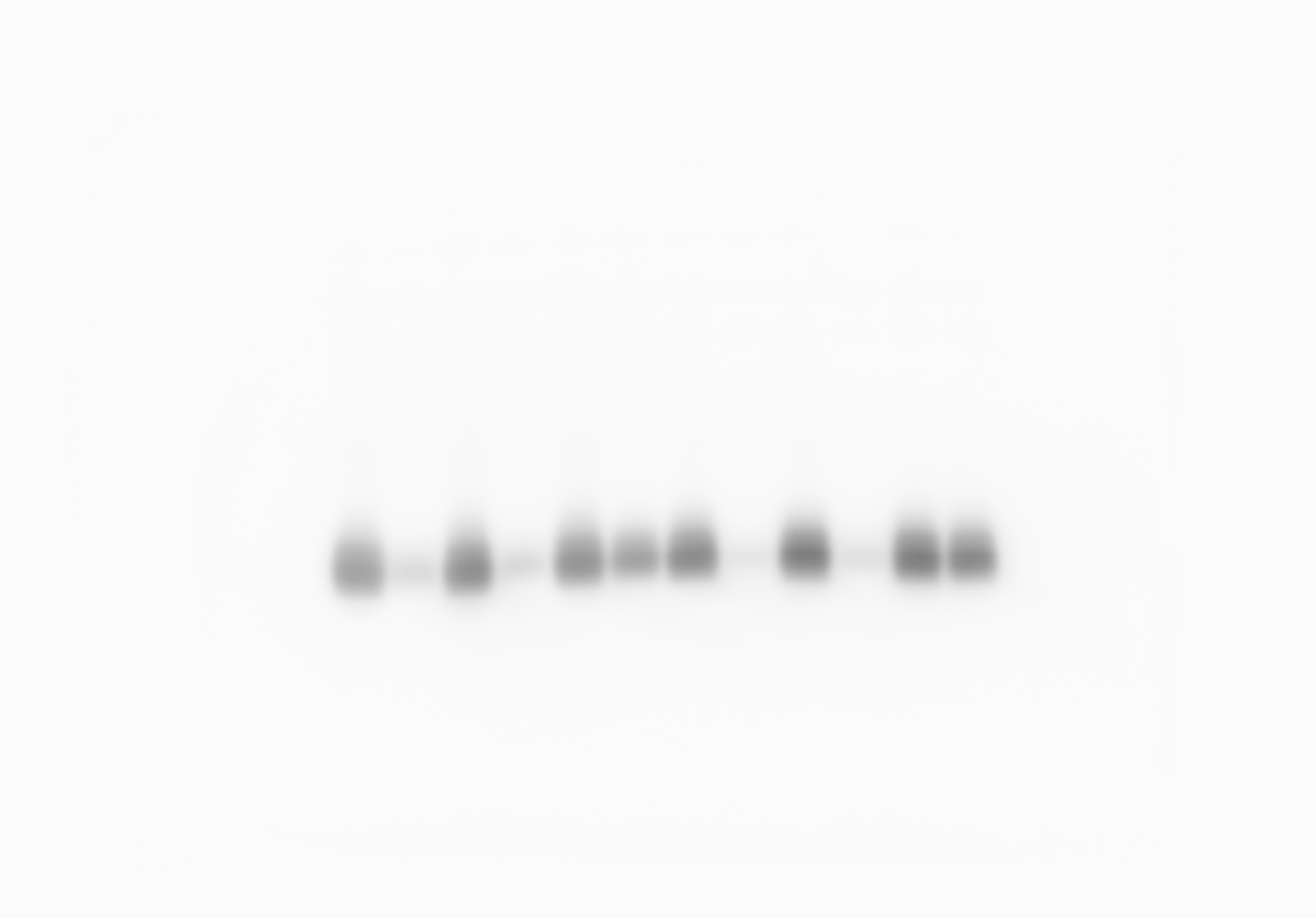

Figure 5; library R, replicate 3; TIFF

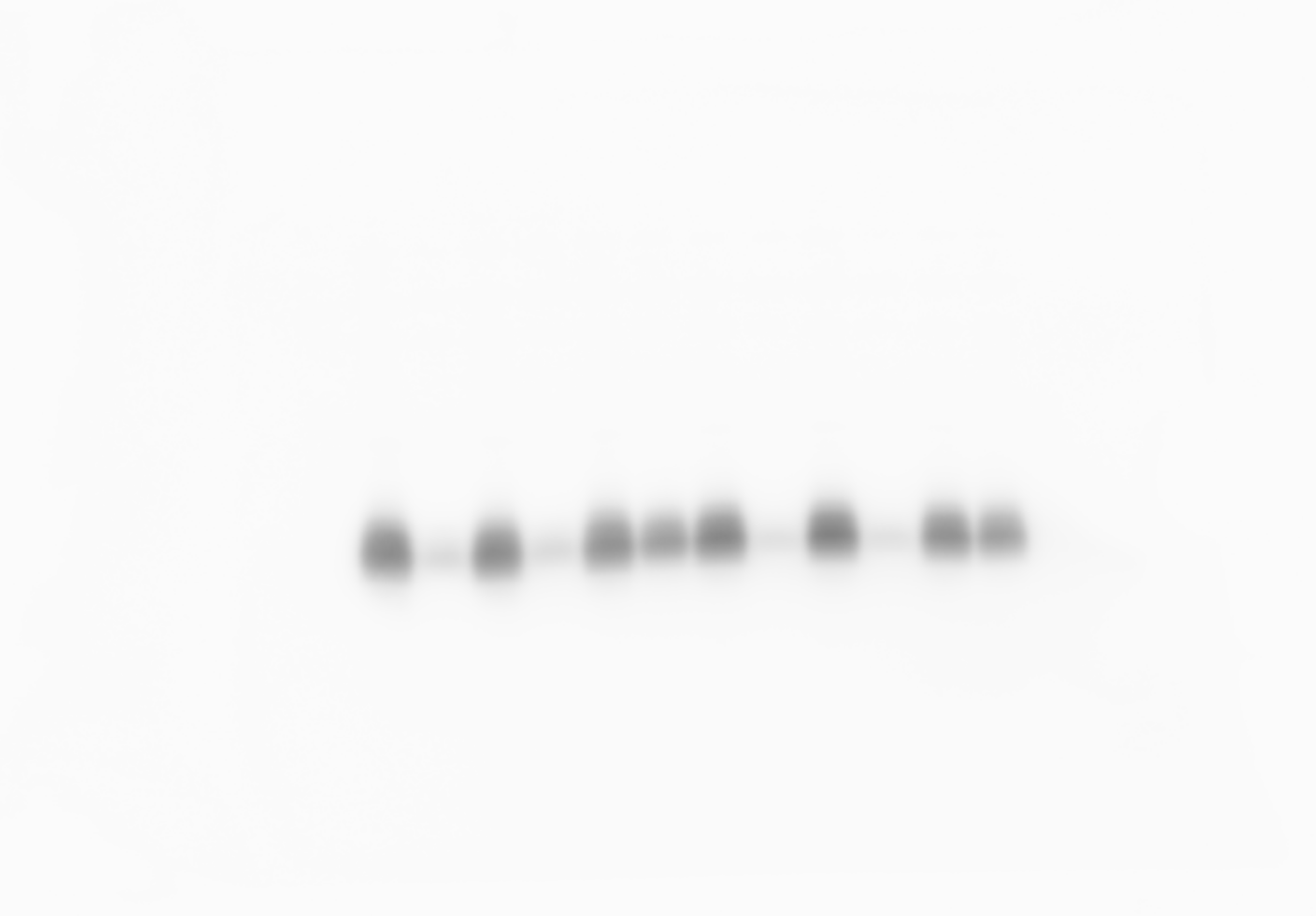

Supplement: Source Data Fig. 5 — Unprocessed western blots. [file 41559_2023_2010_MOESM4_ESM.pdf]
